# Walking with blood flow restriction on lower limb muscles post-ACL reconstruction: A within-subject trial

**DOI:** 10.1371/journal.pone.0333200

**Published:** 2025-10-08

**Authors:** Letícia Pophal Cutisque, John Gerard Buckley, André Luiz Felix Rodacki

**Affiliations:** 1 Department of Physical Education, Federal University of Paraná, Curitiba, Brazil; 2 School of Engineering. University of Bradford, Bradford, West Yorkshire, United Kingdom; Erzurum Technical University: Erzurum Teknik Universitesi, TÜRKIYE

## Abstract

**Background:**

Individuals recovering from anterior cruciate ligament (ACL) reconstruction often exhibit persistent strength deficits that can impair function and delay return to participation in sport and/or physical activity. Blood flow restriction (BFR) training has emerged as a promising strategy to enhance muscle adaptations using low-load exercises, but its effectiveness when combined with walking training in this population remains unclear.

**Objective:**

To determine the effectiveness of a walking program combined with BFR on muscle strength and thickness at the ankle and knee in patients with ACL reconstruction.

**Methods:**

This within-subject clinical trial included 40 adults (27.1 ± 7.3 years; 21 females, 19 males) who had undergone ACL reconstruction at least 6 months prior and presented with a ≥ 10% strength deficit in the knee extensor muscles of the operated limb. Participants completed a 12-week progressive walking program, with duration (from 12 to 20 minutes) and speed (from 1.25 to 2.00 m.s^-1^) being progressively increased over the 12 weeks. BFR at 90% occlusion pressure was applied to the weaker limb (WL), while the contralateral limb served as control (CL). The intervention included one supervised and two home-based walking sessions per week. Post-intervention outcomes involved determining changes from baseline in peak torque (PT) of ankle and knee extensors and flexors, as well as muscle thickness (MT) of the gastrocnemius medialis (GM), tibialis anterior (TA), vastus lateralis (VL), and semitendinosus (ST).

**Results:**

Ankle plantarflexor PT increased in both limbs (WL = 51.6%; CL = 11.7%), while dorsiflexor PT increased slightly in the WL (5%). GM thickness increased in both limbs (WL = 1.1%; CL = 0.5%), with no changes in TA. For the knee extensors and flexors, PT increased in both limbs (WL = 33.1% and 25.7%; CL = 14.9% and 8.9%). No significant MT changes were observed in VL or ST.

**Conclusions:**

A progressive walking program with BFR was associated with increases in lower limb strength and modest MT changes in individuals post-ACL reconstruction. These findings suggest that this intervention may represent a safe and potentially efficient adjunct to post-ACL rehabilitation. **Trial registration:** RBR-5gqgs99. *https://ensaiosclinicos.gov.br/rg/RBR-5gqgs99*.

## 1. Introduction

Each year, over 2 million anterior cruciate ligament injuries (ACL) occur worldwide, with reconstructive surgery commonly recommended for young and active individuals. This procedure is frequently performed to restore mechanical stability, facilitate return to competitive sports activities, and prevent secondary injuries [1]. Despite targeted rehabilitation efforts, significant deficits in muscle strength and endurance, particularly in the muscles surrounding the knee joint, often persist long after surgery due to muscle atrophy and neuromuscular inhibition [2–4]. Recent evidence indicates that approximately 35% of athletes fail to return to their pre-injury level of sport within two years after ACL reconstruction [5]. Notably, half of these individuals report the ACL injury as the main reason for their reduced level of physical activity [5,6].

These impairments lead to altered movement patterns in the affected limb, increasing the risk of early-onset osteoarthritis and other knee conditions [7–9]. Reductions in muscle strength, primarily from the disuse of the thigh muscles acting across the knee, are a significant concern, as the lengthy and challenging rehabilitation process often delays regaining pre-injury muscle strength. This compromises lower limb function and ultimately affects the patient’s overall life quality [7–9].

The intermediate postoperative period after ACL reconstruction, typically ranging from 3 to 6 months post-surgery, corresponds to the conclusion of the proliferative phase and the beginning of graft ligamentization [1]. Although some degree of graft maturation occurs during this time, complete biological and mechanical healing extends well beyond six months. Despite continuous advancements in surgical techniques and rehabilitation strategies, functional outcomes following ACL reconstruction often remain suboptimal [1]. There is limited consensus on the optimal components of ACL rehabilitation, reflecting a lack of robust evidence underpinning guidelines and significant variability across rehabilitation protocols [3]. Current guidelines suggest that ACL patients should perform exercises at intensities ranging from 60% to 100% of their one-repetition maximum (1RM) to enhance strength, explosiveness, and endurance [1]. However, postoperative patients often face limitations in exercising with high loads [2]. In the early stages of rehabilitation following ACL reconstruction, high-intensity exercises (> 70% of 1RM) are contraindicated, and hence more cautious approaches should be considered [1,2].

Given these challenges, exploring new rehabilitation protocols for ACL reconstruction becomes essential to facilitate fast and effective recovery while avoiding detrimental effects, especially in the initial weeks following surgery. Therefore, strategies that enhance muscle strength and mass, as well as reduce asymmetry between limbs, are of significant clinical interest [2,10,11]. Blood flow restriction training has emerged as an alternative method to mitigate the limitations associated with high-intensity training protocols [10,12]. Blood flow restriction involves using inflatable cuffs or wraps placed proximally on the limbs during exercise to occlude arterial blood flow and restrict venous return [13,14]. The method includes exercising at very-light and light intensities (from 20–30% 1RM) [12–14]. Several studies have demonstrated that BFR training effectively promotes strength gains and muscle mass increases, with adaptations comparable to traditional high-intensity training, despite using low-load resistance exercises [13–16]. It is believed that BFR training promotes significant strength gains through a combination of neuromuscular and metabolic mechanisms/adaptations [1,2]. Although these mechanisms have been suggested in previous literature, they were not directly assessed in the present study and are presented here as theoretical explanations. Specifically, this may involve a hypoxic environment induced by vascular occlusion, which accelerates the recruitment of fast-twitch muscle fibers – typically engaged under high loads or fatigue [17,18].

BFR therapy has been shown to support healing following ACL reconstruction [1], and may improve recovery and functional outcomes, as low loads can minimize injury risk and ensure joint and graft safety, while also contributing to postoperative strengthening. Exercises performed under BFR may also mitigate some disadvantages of traditional resistance training, including pain reduction, improved physical function, and enhanced quality of life [1].

Other studies, involving participants that haven’t undergone ACL reconstruction, have used exercise protocols involving the association of BFR and walking, and have reported strength and muscle hypertrophy gains [19–23] in addition to physical function benefits [24]. To date, no studies have investigated whether a training protocol combining walking and BFR induces improvements in strength and muscle mass after ACL reconstruction. Therefore, this study was designed to determine whether a 12-week training program combining BFR and walking, produces muscular adaptations (i.e., increases in muscle strength and thickness, specifically in the ankle and knee muscles), in patients in the mid-term postoperative phase following ACL reconstruction.

## 2. Materials and methods

### 2.1. Participants

This within-subjects clinical trial was conducted in adults who had undergone ACL reconstruction between 6 and 24 months prior to enrolment in the study. The recruitment period began on November 28, 2024, and ended on April 3, 2025. All participants were individually assessed, and those who met the eligibility criteria were invited to participate. They received detailed verbal and written information about the study objectives and procedures. Those who agreed to participate signed an informed consent form. All study procedures were reviewed and approved by the University’s Ethics Committee (approval number: 80899524.0.0000.0102). Participants who consented were enrolled in a training program in the following week, resulting in a rolling admission process. Each participant was evaluated prior to starting their training and re-evaluated after completing 12 weeks of training. Participants observed a 48-hour rest period after their final training session, during which they were instructed to refrain from any physical activity. Post-intervention assessments were then scheduled, with a maximum interval of five days between the final training session and the assessment. The rights and well-being of all participants were protected throughout the entire study.

Sample size was estimated using G*Power software (University of Düsseldorf, Germany). Considering the within-subject design of the study, a repeated-measures analysis of variance (ANOVA) was selected, with two factors: time (pre- and post-intervention) and segment (WL vs. CL), both measured within the same participants. The following parameters were adopted: effect size (ES) = 0.30, an error probability = 0.05, power = 0.90, number of groups = 1, number of measurements = 2 (pre- and post-intervention), correlation among repeated measures = 0.5, and non-sphericity correction = 1. Considering these parameters, the estimated sample size was 32 participants. To account for possible dropouts or missing data, a 20% was added, resulting in a final required sample size of 38 participants.

The eligibility criteria included participants aged between 18 and 59 years, with (i) a history of unilateral ACL reconstruction (from 6 to 24 months post-surgery); (ii) the presence of clinically relevant muscle strength weakness (in the reconstructed limb), defined as a ≥ 10% strength deficit in the knee extensor muscles compared to contralateral limb (mean: 17.65 [SD: 11.24%]), which ensured a functionally homogeneous sample regardless of postoperative time; (iii) medical clearance (granted by a physician) to carry out the training protocol and, (iv) no history of surgery on the contralateral knee. The exclusion criteria were: (i) having a lower limb musculoskeletal injury that could substantially compromise functional performance or interfere with the protocol (e.g., ACL re-injury; meniscal tear, ankle sprain), (ii) inability to perform and conclude all procedures; and, (iii) presented with resting systolic blood pressure ≥ 140 mmHg or diastolic blood pressure ≥ 90 mmHg, regardless of whether they used antihypertensive medication or not. This criterion was adopted to ensure safety during BFR training, which, although associated with lower cardiovascular strain than high-intensity resistance training, may still elicit acute elevations in blood pressure. In line with safety guidelines [13], participants were also excluded if they presented more than one risk factor for thromboembolic events, including (iv) obesity (BMI ≥ 30 kg.m^-2^), (v) Crohn’s or Inflammatory Bowel Disease, (vi) past fractures of the hip, pelvis or femur, (vii) major surgeries performed within the past six months, particularly those associated with an increased risk of thromboembolic events (e.g., abdominal or vascular procedures), (viii) diagnosed with varicose veins, (ix) family history of deep vein thrombosis or pulmonary embolism, (x) currently using oral contraceptive (xi), or smoked on a daily basis [13].

**Fig 1:**
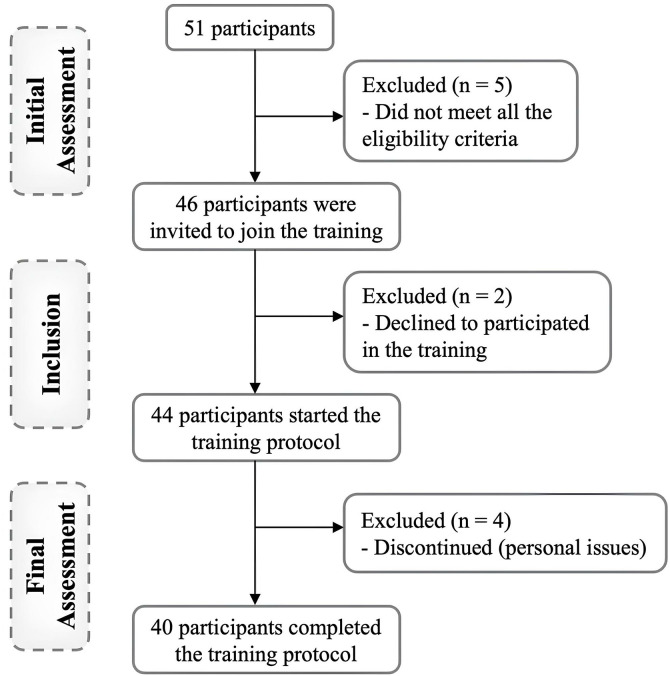
Schematic representation of participants’ recruitment and allocation.

In this study, BFR was applied to the weaker lower limb, referred to as the weaker limb (WL), while the stronger lower limb served as the control limb (CL). Notably, although the CL was designated as a control, it was actively involved in the walking activity alongside the WL. Most participants presented visible surgical scars, which meant assessors could not be blinded regarding which limb was the WL and which was the CL. However, to minimize potential bias, trained researchers conducted the evaluations using a standardized protocol, with each evaluator assigned to a specific task. A schematic representation of participants’ recruitment and allocation is provided in Fig 1.

### 2.2. Outcome measurements

#### 2.2.1. *Personal and health-related data.*

Outcome measures were assessed at baseline (1 week after enrolment) and post-intervention (2 days after completion of the 12-week training program). Information regarding age, gender, anthropometric data (stature, body mass, and body mass index), self-reported physical activity (PA) level, and sedentary behavior (SB) was assessed. Additional data related to the ACL surgery, including time since surgery, duration of postoperative rehabilitation, and graft type, were also collected; however, most participants were unable to provide accurate information on graft type. Therefore, these data were not included in the analysis.

#### 2.2.2. *Muscle strength.*

The isometric peak torque of the ankle (plantarflexors and dorsiflexors) and knee (extensors and flexors) were assessed using an isokinetic dynamometer (Biodex Medical Systems 3; Inc. Shirley. NY. USA). The participants were instructed not to perform lower limb exercises 48 hours before the assessment. For the assessment of the ankle muscles, the rotation axis of the device was aligned with the lateral malleolus of the ankle (the anatomical axis of rotation of the ankle). The knee muscles were assessed with the device’s rotation axis aligned with the knee’s lateral epicondyle (i.e., the anatomical axis of rotation of the knee). The protocol consisted of three warm-up trials and three maximal isometric voluntary contractions (MIVC), each lasting 5s, with a 120s interval between repetitions. During attempts, participants received verbal incentives. The average PT of the three attempts was used for further analysis. The reliability of isometric strength assessments of both ankle and knee muscle groups has been previously established. In adult populations, test-retest intraclass correlation coefficients (ICCs) of 0.88 for plantarflexors and 0.98 for dorsiflexors have been reported [25], along with ICCs of approximately 0.90 for both knee extensors and flexors, indicating high to excellent measurement consistency across all muscle groups [26].

#### 2.2.3. *Muscle thickness.*

**Fig 2:**
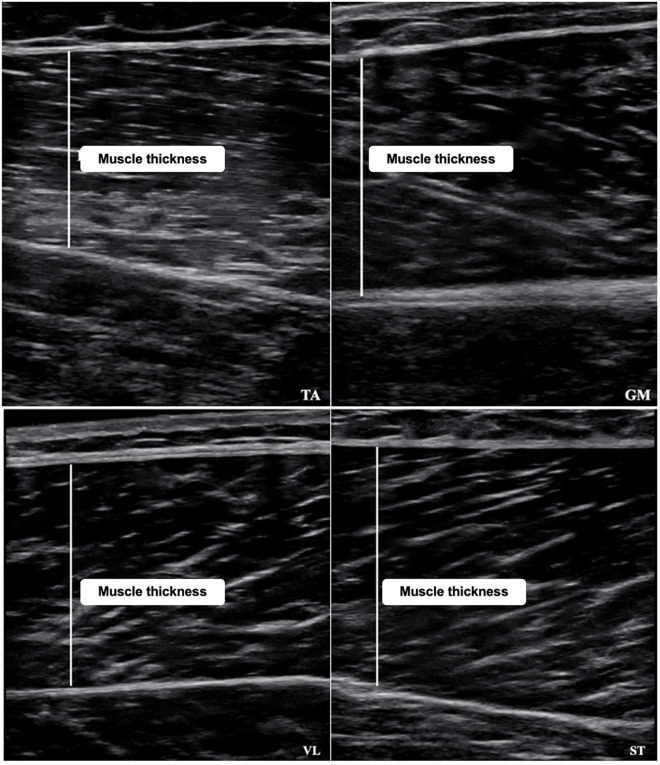
Muscle thickness (MT) images of the Tibialis Anterior (TA – upper left), Gastrocnemius Medialis (GM – upper right), Vastus Lateralis (VL – lower left), and Semitendinosus (ST – lower right).

Muscle thickness (MT) was used as a proxy for muscle hypertrophy [27]. MT was estimated as the distance between the superficial and deep aponeuroses [28,29] using a portable Mode-B Ultrasound (System 1.0 Konica Minolta®, model Sonimage HS1) equipped with an 11 kHz linear transducer. The participants were instructed to refrain from lower limb exercises 48 h before testing [30,31]. A thick layer of water-soluble transmitting gel was applied to the transducer to provide acoustic contact without depressing the skin. The transducer was positioned perpendicular along the muscle axis. The scanning depth was kept constant at 5 cm and adjusted to encompass a sufficient muscle area in participants with prominent subcutaneous fat. All participants were lying prone and instructed to relax their muscles as much as possible. The measurement sites were marked with a permanent skin marker (reinforced weekly) to improve consistency. Three images of each muscle were recorded, and the average was used for analysis purposes. The MT of the ankle plantarflexor was conducted on the gastrocnemius medialis (GM), located at 30% of the distance from the popliteal fossa to the medial malleolus [32]. The ankle dorsiflexor measurements were performed on the tibialis anterior (TA), approximately at 20% of the distance from the fibular head to the medial malleolus [33]. The knee extensor assessment was carried out on the vastus lateralis (VL) at the midpoint (50%) between the femoral greater trochanter and the proximal-lateral edge of the patella [28,29]. The thickness of the knee flexor was measured on the semitendinosus muscle (ST), at 50% of the distance between the ischial tuberosity and the medial tibial condyle [28,29]. Fig 2 presents a representative image of the GM, TA, VL, and ST assessments. A pilot study involving assessment of 5 participants revealed a high intraclass correlation coefficient (ICC) of the MT (ICC_GM_ = 0.93; ICC_TA_ = 0.94; ICC_VL_ = 0.96; ICC_ST_ = 0.95) after two consecutive visits performed 48 hours apart.

### 2.3. *Intervention*

Although the initial protocol submitted to the ethics committee included the possibility of comparing blood flow restriction (BFR) training with traditional resistance exercise, the latter intervention was not implemented. Thus, the primary objective of the present study was to investigate the intra-individual effects of BFR walking exercise on neuromuscular outcomes in the weaker limb following anterior cruciate ligament (ACL) reconstruction. Thus, a within-subjects clinical trial design was adopted in which each participant served as their own control, with the weaker limb (WL) receiving the BFR intervention and the contralateral, stronger limb acting as the control condition (CL).

A familiarization session was conducted before the start of the training. It included a clinical assessment for medical clearance and a detailed explanation of the procedures used to induce partial vascular restriction. Recommendations for BFR pressure vary from 40–90% of the pressure necessary to cause complete arterial occlusion, suggesting that an individualized pressure is recommended to produce the desired effects while minimizing unnecessary risks [11]. The BFR pressure was individually determined using a large sphygmomanometer (Premium®, model BR 20D; 18 cm in width) and a vascular Doppler device (Martec®, model DV600). A nylon cuff was placed at the most proximal portion of the weaker limb (i.e., at the gluteal fold) [34], and the Doppler probe was positioned over the tibial anterior artery. Cuff pressure was gradually increased in 2 mmHg increments until the auscultatory pulse signal could no longer be detected [35]. The lowest pressure at which no pulse was observed was defined as the arterial occlusion pressure [13,14], and 90% of this value was adopted for training purposes [10,36,37].

Participants engaged in the intervention three times per week for 12 weeks (36 sessions), consisting of one in-person and two home-based sessions. In-person sessions were conducted in small groups at the Biomechanics Laboratory of the Department of Physical Education, and were supervised by two trained and experienced researchers, both of whom were licensed physiotherapists. Group sessions were scheduled at different times slots to ensure adequate monitoring and individualized guidance. During these sessions, researchers ensured correct cuff placement, precise pressure adjustment, and standardization of walking cadence using a metronome mobile app (Cifra club®). During supervised training, the app was operated by the researchers and connected to a portable speaker so that the cadence was clearly audible to all participants. Walking was performed back and forth along a ~ 20-m flat path along the laboratory floor.

Participants replicated the procedures during the home-based sessions using the same cuffs and manometers and were instructed to monitor and maintain the prescribed 90% of occlusion pressure. For these sessions, participants were advised to perform the walking protocol using any flat and open area available to them – such as sidewalks, driveways, sports courts, or hallways – ensuring they maintained the total session duration and prescribed cadence, even if the path length was shorter than 20 m. Cadence was controlled by using the same metronome app on their personal devices to match the walking speed taught during supervised sessions.

After each session, the cuff was deflated, and participants performed five minutes of unrestricted walking to promote venous return. The training sessions utilized an individualized BFR pressure, corresponding to 90% of each participant’s arterial occlusion pressure, which resulted in a group mean applied pressure of 125.8 ± 6.0 mmHg. Participants reported the training sessions (over the 12 week period) to have, a perceived exertion of very light to light intensity (10.5 [2.1 a.u.]) [38], and experienced light to moderate discomfort (3.5 [2.0 a.u.]) [39–41]. In-person sessions provided an opportunity for researchers to control for any noticeable compensatory movements participants had when walking, offer guidance, and monitor participants’ vital signs. Twelve in-person sessions were held, with a high adherence rate (91%), ensuring that participants completed at least 11 sessions. The adherence for home-based sessions was 87% and corresponded to a minimum of 21 sessions. Overall adherence to the training program was 90%, with the total number of completed sessions ranging from 28 to 36.

Completion of a weekly training diary was mandatory, and included recording the completion of each session, the Borg RPE and discomfort ratings (0–10 scale) induced, and any possible adverse events experienced. Participants were offered two options for completing the diary: a physical version, which was returned to the researchers during in-person sessions (with a new copy provided at each meeting), and an online version, which participants were instructed to complete immediately after each home-based session. Both formats contained the same information. The dual-format option aimed to accommodate participants who might face difficulties using digital tools. In cases where neither the physical diary was returned nor the online questionnaire completed, the research team contacted the participant within the same week to verify adherence and collect the missing data.

To support training adherence and engagement, a communication group was created using a mobile messaging application, which included all participants and researchers. On the day of each home-based session, reminder messages were sent encouraging participants to complete the session and to then share photos or videos as proof of execution. Although sharing media was optional, many participants voluntarily posted content, which served as motivation to themselves and others and increased engagement. This group remained active throughout the 12-week period, ensuring continuous contact and support. At the conclusion of the intervention, each participant received a personalized report summarizing their initial and final assessments of muscle strength and thickness, presented using bar graphs to facilitate understanding and interpretation of their progress.

The training program followed a six-phase progression model, gradually increasing walking time, cadence, speed, and distance. Details regarding the duration and structure of each phase are presented in Table 1. To minimize the influence of external physical activities and ensure the internal validity of the findings, participants were instructed not to initiate any new exercise programs or discontinue any current forms of physical activity during the 12-week training/intervention period. Participants had previously completed variable durations of rehabilitation (mean = 6.6 ± 4.0 months) but were no longer engaged in structured rehabilitation programs at the time of enrollment. All participants were advised to maintain their usual daily routines throughout the study period and to report any changes.

**Table 1:** Blood flow restricted training protocol parameters across 12 weeks (36 sessions).

Phases	Weeks	Time	Cadence	Speed	Distance	Duration
**Phase 1**	Week 1	12 min	110 steps.min^-1^	1.25 m.s^-1^	900m	2 weeks
	Week 2					
**Phase 2**	Week 3	14 min	110 steps.min^-1^	1.30 m.s^-1^	1100 m	1 week
**Phase 3**	Week 4	16 min	110 steps.min^-1^	1.36 m.s^-1^	1300 m	1 week
**Phase 4**	Week 5	18 min	115 steps.min^-1^	1.47 m.s^-1^	1600 m	1 week
**Phase 5**	Week 6	20 min	115 steps.min^-1^	1.91 m.s^-1^	2300 m	3 weeks
	Week 7					
	Week 8					
**Phase 6**	Week 9	20 min	120 steps.min^-1^	2.00 m.s^-1^	2400 m	4 weeks
	Week 10					
	Week 11					
	Week 12					

**Footnote:** The training progression was based on gradual increases in walking time, cadence, and speed, allowing participants to adapt progressively to both the physical demands and the sensation of BFR. In the initial phase (weeks 1−2), we adopted a conservative walking speed (1.25 m.s-1) with relatively low cadence (110 steps.min-1) to allow familiarization with the BFR stimulus and minimize discomfort. From week 3 onward, the walking time and speed were progressively increased each week, while cadence remained stable, allowing participants to gradually tolerate longer durations and higher intensities. At week 5, cadence was increased to 115 steps.min-1 and maintained during Phase 5. Finally, during the last 4 weeks (phase 6), we implemented the highest cadence (120 steps.min-1) and fastest walking speed (2.00 m.s-1) for 20 minutes, maximizing the training effect. Importantly, participants walked for 20 minutes in 7 of the 12 weeks, consistent with BFR principles that emphasize metabolic stress under low loads. Therefore, the progression was designed to respect safety and tolerance thresholds while gradually increasing training volume and metabolic demand, optimizing adaptation while maintaining comfort and adherence. Finally, increasing the training load (gradual increases in walking time, cadence, and speed) was implemented on purpose, since most protocols use a fixed load throughout the training period.

### 2.4. Data analysis

Descriptive statistics were used to characterize the sample. Dependent (paired) t-tests was used to analyze the differences between pre- and post-intervention regarding participants’ personal characteristics (secondary outcomes). Linear Mixed Model ANOVAs were used to analyze changes in muscle strength (PT) and muscle thickness (MT), considering time (pre vs. post), limb (WL vs. CL), and the interaction time x limb as fixed effects. We initially ran the statistical model with ‘gender’ as a fixed factor, but as this analysis indicated no significant differences between males and females, the final model used consider the sample group as a whole. Participants were included as a random intercept to account for within-subject repeated measures. Bonferroni adjusted post-hoc comparisons identified where differences occurred between limbs before and after the intervention. Deltas (Δ) were calculated to represent pre-post changes. To determine the clinical relevance of the observed changes, the Minimal Detectable Change (MDC) at the 95% confidence level (MDC₉₅) was calculated for each outcome variable using the following formula: $MDC=\text{1}.\text{9}\text{6}\;x\;\sqrt{\text{2}}\;x\;SEM$. The Standard Error of Measurement (SEM) was estimated from baseline standard deviations (SD) and intra-class correlation coefficients (ICCs), following the equation: $SEM=SD\;x\;\sqrt{}(\text{1}-ICC)$. For muscle strength variables, ICCs were obtained from previously published studies [26]. For muscle thickness variables, ICCs were derived from reliability analyses conducted within the current study. The MDC values were then compared with the observed pre-to-post changes (Δ) in both the weaker and control limbs to assess whether the magnitude of change exceeded the threshold of measurement error and thus could be considered clinically meaningful. Additionally, paired-sample Cohen’s d effect sizes were calculated to quantify the magnitude of pre-to-post-intervention changes for each limb and outcome, using the standard deviation of the difference scores. The interpretation of d values followed the conventional thresholds: d < 0.2 was considered a trivial effect, 0.2 ≤ d < 0.5 small, 0.5 ≤ d < 0.8 moderate, and d ≥ 0.8 large [42]. There was no missing data. Analyses were performed in SPSS (version 25), with statistical significance set at p < 0.05.

## 3. Results

Forty participants (21 females, 52.5%; 19 males, 47.5%) were assessed before and after the intervention. Although the eligibility criteria included participants aged 18–59 years, the actual sample ranged from 18 to 43 years (27.1 [7.3 years]), which was not intentional but reflects the participants who volunteered and met the inclusion criteria. All participants had already been cleared by their treating physician or physiotherapist to return to their usual physical activities. The results showed a discrete increase in body mass after the training period (77.2 vs. 78.4 kg; p < 0.001; ES = 0.6), with a reduction observed in sedentary behavior (from 46.9 to 40.6 hours.week^-1^, p = 0.007; ES = 0.5). Additionally, a significant reduction was found in systolic blood pressure after training, which decreased from 121.3 to 117.3 mmHg (p < 0.001; ES = 0.6), while no changes were observed in diastolic blood pressure. None of the other physical measures differed after training (p > 0.05). The participants’ characteristics are presented in Table 2.

**Table 2:** Physical and health-related parameters assessed before (PRE) and after (POST) 12 weeks of walking with blood flow restriction (n = 40).

Characteristics	Pre-intervention Mean (SD)	95% CI		Post-intervention Mean (SD)	95% CI		p value	ES	Δ %
		Inf.	Sup.		Inf.	Sup.			
Body mass (kg)	77.2 (11.6)	73.5	80.9	78.4 (12.2)	74.5	82.3	**< 0.001**	0.6	1.6
Body mass index (BMI, kg.m^-2^)	26.2 (2.7)	25.3	27.0	26.4 (2.8)	25.5	27.3	0.262	0.2	0.8
Blood pressure systolic (BPS, mmHg)	121.3 (6.7)	119.1	123.4	117.3 (8.0)	114.8	119.9	**< 0.001**	0.6	−3.3
Blood pressure diastolic (BPD, mmHg)	78.5 (5.9)	76.6	80.4	77.6 (5.4)	75.8	79.3	0.136	0.2	−1.2
Heart rate (HR, bpm)	72.7 (7.6)	70.3	75.2	71.1 (6.6)	68.9	73.2	0.097	0.3	−2.2
Physical activity (PA, min.week^-1^)	300.4 (179.3)	243.04	357.71	267.6 (197.6)	204.4	330.8	0.395	0.1	−10.9
Sedentary behavior (SB, hours.week^-1^)	46.9 (16.6)	41.5	52.2	40.6 (15.8)	35.6	45.7	**0.007**	0.5	−13.4

Abbreviations: SD, standard deviation; CI, confidence interval.

Bold text indicates statistically significant.

### Muscle strength (PT) of the ankle

After the intervention, the weaker limb (WL) exhibited muscle strength (PT) increases in the ankle plantarflexors (73.4 N.m vs. 111.3 N.m; Δ = 51.6%; p < 0.001). The control limb (CL) also experienced strength increases but to a lesser extent (96.5 N.m vs. 107.8 N.m; Δ = 11.7%; p = 0.014). Notably, the strength gains were significantly greater in the WL compared to the CL, as confirmed by a significant interaction effect (p < 0.001), indicating that the limbs responded differently to the intervention.

Dorsiflexion PT differed between limbs (p < 0.001) and there was a significant interaction between limb and time (p = 0.003) but no main effect of time (p = 0.685). This indicates that changes (in dorsiflexion PT) over time differed between the WL and the CL. Specifically, the WL demonstrated a small increase in dorsiflexion PT (Δ = 5.9) from pre- to post-intervention, while the CL exhibited a small decrease (Δ = –3.2); however, post-hoc analysis indicates that these differences were non-significant (p > 0.33).

### Muscle thickness (MT) of the lower leg

Modest increases in muscle thickness (MT) of the gastrocnemius medialis (GM) were observed in both limbs (WL and CL) following the intervention (WL = 1.87 cm to 1.89 cm; CL = 1.91 cm to 1.92 cm; p < 0.001). However, there were no significant differences between limbs, and no interaction effects (p > 0.05). This indicates that the increase in GM thickness occurred uniformly across limbs, regardless of the intervention. No significant differences were found in MT of the tibialis anterior (TA) between limbs or pre- and post-intervention (p > 0.05).

### Interlimb differences in ankle muscle strength (PT) and thickness (MT)

Baseline asymmetries between the WL and CL were evident in plantarflexor and dorsiflexor PT, with the WL presenting significantly lower strength than the CL (plantarflexors = 73.4 N.m vs. 96.5 N.m, Δ = −23.9%; dorsiflexors = 34.0 N.m vs. 37.8 N.m, Δ = −10%; p < 0.001). Following the intervention, no significant differences were found between the limbs in either muscle (p > 0.05), suggesting restoration of interlimb strength symmetry had been achieved. Additionally, no significant differences in MT were observed between the limbs either before or after the intervention (p > 0.05). Detailed data of the PT and MT of the ankle plantarflexors and dorsiflexors are presented in Table 3.

**Table 3:** Muscle characteristics (strength and muscle thickness) of the weaker and control limbs of the ankle plantarflexor and dorsiflexor muscles before (PRE) and after (POST) the BFR walking training intervention.

Control limb (CL)											
Characteristics	Pre-intervention Mean (SD)	SE	95% CI		Post-intervention Mean (SD)	SE	95% CI		ES	Δ %	MDC
			Inf.	Sup.			Inf.	Sup.			
**Muscle strength (PT)**											
Ankle plantarflexor (GM)(N.m)	96.53 (26.45)	4.21	88.07	104.99	107.79 (28.01)	4.34	98.83	116.74	0.5	11.7^a,b,†^	25.4
Ankle dorsiflexor (TA) (N.m)	37.77 (8.74)	1.51	34.98	40.57	36.55 (11.20)	1.67	32.96	40.13	0.2	−3.2^b,†^	3.4
**Muscle thickness (MT)**											
Ankle plantarflexor (GM) (cm)	1.91 (0.28)	0.04	1.82	2.00	1.92 (0.28)	0.04	1.83	2.01	0.2	0.5	0.2
Ankle dorsiflexor (TA) (cm)	1.92 (0.47)	0.07	1.77	2.07	1.91 (0.34)	0.06	1.80	2.02	0.1	−0.5	0.3
**Weaker limb (WL)**											
**Characteristics**	**Pre-intervention Mean (SD)**	**SE**	**95% CI**		**Post-intervention Mean (SD)**	**SE**	**95% CI**		**ES**	**Δ %**	**MDC**
			**Inf.**	**Sup.**			**Inf.**	**Sup.**			
**Muscle strength (PT)**											
Ankle plantarflexor (GM)(N.m)	73.42 (23.43)	4.08	65.93	80.91	111.30 (27.48)	4.06	102.51	120.09	1.7	51.6^a,b,†^	22.5
Ankle dorsiflexor (TA) (N.m)	34.01 (9.82)	1.49	30.87	37.16	36.02 (10.43)	1.70	32.69	39.36	0.3	5.9^b,†^	3.9
**Muscle thickness (MT)**											
Ankle plantarflexor (GM) (cm)	1.89 (0.29)	0.05	1.80	1.98	1.90 (0.28)	0.05	1.81	1.99	0.2	0.5	0.2
Ankle dorsiflexor (TA) (cm)	1.99 (0.43)	0.07	1.85	2.13	2.02 (0.35)	0.06	1.91	2.13	0.1	1.5	0.3

Abbreviations: SD, standard deviation; SE, standard error; CI, confidence interval; ES, effect size; Δ %, delta; MDC, minimal detectable change; CL, control limb; WL, weaker limb; PT, peak torque; GM, gastrocnemius medialis; TA, tibialis anterior; MT, muscle thickness; ^a^within-segment difference (pre- vs. post-intervention); ^b^between-segment difference (CL vs. WL); ^†^limb *x* time interaction.

### Muscle strength (PT) of the knee

The PT of the knee extensors increased significantly in both limbs following the intervention, with the WL showing a greater relative gain (147.8 N.m vs. 195.9 N.m; Δ = 33.1%; p < 0.001) compared to the CL (173.5 N.m vs. 199.3 N.m; Δ = 14.9%; p < 0.001). Significant effects were found for time, limb, and their interaction (p < 0.001), suggesting that the intervention elicited a more pronounced response in the WL. Similar results were observed for the knee flexors, with the WL exhibiting greater relative gain (93.5 N.m vs. 117.5 N.m; Δ = 25%), compared to the CL (100.2 N.m vs. 109.0 N.m; Δ = 8.9%) (Table 4).

**Table 4:** Muscle characteristics (strength and muscle thickness) on the weaker and control limbs of the knee extensor and flexor muscles before (PRE) and after (POST) the BFR walking training intervention.

Control limb (CL)											
Characteristics	Pre-intervention Mean (SD)	SE	95% CI		Post-intervention Mean (SD)	SE	95% CI		ES	Δ %	MDC
			Inf.	Sup.			Inf.	Sup.			
**Muscle strength (PT)**											
Knee extensor (VL) (N.m)	173.53 (49.08)	7.83	157.83	189.22	199.32 (58.01)	8.74	180.77	217.88	0.6	14.86 ^a,b,†^	43.02
Knee flexor (ST) (N.m)	100.18 (31.24)	4.94	90.19	110.17	109.01 (31.81)	5.05	98.84	119.18	0.7	8.90^a,†^	27.38
**Muscle thickness (MT)**											
Knee extensor (VL) (cm)	2.13 (0.30)	0.05	2.03	2.22	2.12 (0.30)	0.05	2.02	2.21	0.1	−0.47	0.17
Knee flexor (ST) (cm)	1.93 (0.31)	0.05	1.83	2.03	1.96 (0.30)	0.05	1.86	2.06	0.3	1.55	0.19
**Weaker limb (WL)**											
**Characteristics**	**Pre-intervention Mean (SD)**	**SE**	**95% CI**		**Post-intervention Mean (SD)**	**SE**	**95% CI**		**ES**	**Δ %**	**MDC**
			**Inf.**	**Sup.**			**Inf.**	**Sup.**			
**Muscle strength (PT)**											
Knee extensor (VL) (N.m)	147.17 (51.00)	8.36	130.86	163.48	195.88 (53.16)	8.51	178.88	212.88	1.9	33.11^a,b,†^	44.70
Knee flexor (ST) (N.m)	93.47 (30.93)	4.90	83.58	103.37	117.53 (31.56)	4.97	107.43	127.62	1.8	25.75^a,†^	27.11
**Muscle thickness (MT)**											
Knee extensor (VL) (cm)	2.12 (0.31)	0.05	2.02	2.22	2.13 (0.31)	0.05	2.03	2.23	0.6	0.47	0.20
Knee flexor (ST) (cm)	1.89 (0.32)	0.05	1.79	1.99	1.91 (0.31)	0.05	1.81	2.01	0.4	1.06	0.20

Abbreviations: SD, standard deviation; SE, standard error; CI, confidence interval; ES, effect size; Δ %, delta; MDC, minimal detectable change; CL, control limb; WL, weaker limb; PT, peak torque; VL, vastus lateralis; ST, semitendinosus; MT, muscle thickness; ^a^within-segment difference (pre- vs. post-intervention); ^b^between-segment difference (CL vs. WL); ^†^significant limb *x* time interaction.

### Muscle thickness (MT) of the thigh

No significant changes were observed in the MT of either the vastus lateralis (VL) or the semitendinosus (ST) in both limbs following the intervention (p > 0.05), indicating that training did not induce measurable hypertrophy in these thigh muscles.

### Interlimb differences in knee muscle strength (PT) and thickness (MT)

Before training, the PT difference in the knee extensors between the WL and CL was approximately 26% (p < 0.001), indicating a marked asymmetry. After the intervention, this difference was reduced to about 3% (p > 0.05), suggesting a near full recovery of strength in the WL. In contrast, knee flexor strength showed no significant asymmetry at baseline (difference of 6.7%; p = 0.131), but following the intervention, such difference increased to 8.7% (p = 0.024), indicating a greater response to the training on the CL than on the WL. Regarding MT, no significant interlimb differences were observed for either the VL or ST muscles before or after the training period (p > 0.05). Detailed results are presented in Table 4.

## 4. Discussion

This study was designed to determine whether a 12-week training program involving walking combined with blood flow restriction (BFR) produces muscular adaptations in patients in the mid-term postoperative phase following ACL reconstruction. Specifically, the study focuses on whether BFR walking training leads to improvements in the muscle strength and thickness of the ankle and knee flexors and extensors. This study is the first to implement a walking protocol combined with BFR, utilizing both in-person and remote sessions. The intervention was applied to the weaker limb (WL), with the stronger limb serving as the control (CL). This within-subject design enabled a direct comparison of local effects, reducing the inter-subject variability inherent in control-group trials, where participant characteristics may differ between groups. This study addresses a specific methodological gap in the rehabilitation literature: the lack of accessible, evidence-based BFR walking protocols tailored for patients with ACL reconstruction who have persistent postoperative deficits in muscle strength. There are no previous studies that have applied BFR walking training unilaterally in a structured and progressive manner, nor combined it with a hybrid supervision model –alternating in-person and remote monitoring – to facilitate both safety and adherence. The novelty of the present study lies in demonstrating that a low-load BFR walking protocol, remotely supported and applied exclusively to the weaker limb, was associated with inducing clinically meaningful muscle strength gains. These gains were observed not only in the muscle groups proximal to the reconstructed knee (e.g., quadriceps), which are known to benefit from standard BFR resistance training, but also in distal regions (e.g., gastrocnemius), where muscle adaptations are less frequently reported. Notably, the strength improvements in the occluded limb exceeded those typically attributed to cross-education effects – i.e., the neural adaptations that occur in the contralateral, non—trained limb – highlighting the potential of this BFR walking protocol as a feasible alternative to high-load resistance training, particularly during the early-to-mid-phase rehabilitation following ACL reconstruction.

Recent evidence supports the application of resistance training with BFR in ACL rehabilitation. For example, Li et al. (2023) conducted an 8-week randomized trial comparing quadriceps training with BFR of 40% and 80% AOP in post-ACL reconstruction patients. The 80% AOP group demonstrated approximately ~45% increases in quadriceps strength, along with a ~ 33% increase in muscle thickness of the rectus femoris and vastus intermedius – significantly outperforming both the 40% AOP and non-occlusive control groups [1]. Similarly, Gopinatth et al., (2024) performed a meta-analysis of Randomized Clinical Trials (RCTs) in ACL and knee OA populations and found that 8–12 weeks of resistance training with BFR of ~80% AOP led to moderate improvements in quadriceps strength and clinically meaningful gains in knee-perceptual scores compared to non-BFR rehab [43]. These findings indicate that resistance training with BFR can yield strength and hypertrophy outcomes comparable to – if not exceeding – those of high-load resistance training, with the added advantage of being safe and effective during early rehabilitation when high mechanical loads are contraindicated [1,10]. However, while the benefits of BFR resistance training are well established, there is still limited and inconclusive evidence regarding the efficacy of BFR applied to walking protocols in this population.

Interestingly, despite the absence of external loads, the present study demonstrated significant gains in muscle strength following completion of the 12-week BFR-walking protocol. These improvements may be explained by several contributing factors, one of which is the progressive increase in training intensity across the intervention. Notably, the initial walking speed in the present protocol (1.25 m.s^-1^) was already higher than that adopted by Abe and colleagues, and was progressively increased up to 2.0 m.s^-1^ throughout the program. In contrast, Abe et al. maintained a constant speed of 0.8 m.s^-1^ over the training period [44]. It is therefore plausible that, in the present study, both the greater walking speed and the progressive nature of the intensity led to a higher number of steps per session and, consequently, a greater cumulative neuromuscular stimulus – in comparison to previous work. Moreover, walking at faster speeds is associated with increased mechanical demands, as higher joint torques are required, thereby likely inducing greater muscle activation during each session [45,46]. Such findings may be particularly relevant in the context of ACL rehabilitation, where high-load strength training is often contraindicated during the early phases of recovery [10]. The ability to elicit strength improvements through a progressively intensified BFR-walking protocol – which imposes minimal joint stress – offers a promising strategy for maintaining or enhancing neuromuscular function and graft integrity [10,43]. These results support the growing body of evidence indicating that low-load BFR modalities, including walking, can serve as a viable and safe alternative for ACL-reconstructed patients.

To better understand the potential of BFR-walking for eliciting strength adaptations, especially in clinical populations, previous studies have investigated its effects across different protocols and intensities. Ozaki and colleagues investigated the strength gains and hypertrophic responses associated with BFR walking in sedentary older adults, using four weekly sessions over a 10-week period [20]. They implemented a 20-minute treadmill BFR walking protocol, with the walking speed set to 45% of the participants’ heart rate reserve. Although the actual walking speed was not reported, it was likely considerably lower than that used in the present study. The authors observed moderate increases in knee extension and flexion strength (8.7% and 15.0%, respectively). In comparison, Neto and colleagues applied a BFR-walking protocol in osteoporotic older adults, with a training intensity set at 65% of the maximum heart rate reserve. Although walking speed was not reported, it was individually adjusted to achieve the target heart rate. The protocol consisted of two 15-minute sessions per week for 12 weeks [23]. Although the training load remained constant over the 12 weeks, the higher intensity (65% vs. 45%) likely required a faster walking speed compared to that used by Ozaki [19]. This may partly explain the larger strength gains in the knee extensors observed by Neto et al. (21%), which were closer to those found in the present study (33%).

The present study also demonstrated that 12 weeks of BFR walking was associated with substantial strength increases (25%) in knee flexor muscles comparable to those reported in previous studies [20]. These strength gains however were smaller than those found for the knee extensors, which is in agreement with previous research [47–49]. The magnitude of the relative gains in extensor compared to flexor muscles may be related to the requirements of gait, in which the knee extensors are far more activated than their flexor antagonists [47–49]. For example, when walking at a self-selected speed the vastus medialis muscle is activated through the gait cycle about twice as much as the knee flexors [47–49]. The mean magnitude of activation of the knee flexors (e.g., semitendinosus) during walking has been reported to vary around 10–15% of a maximal voluntary isometric contraction (MVIC) [47–49]. Therefore, BFR walking training may only be associated with strength changes in the muscles/muscle region where a minimum recruitment threshold is achieved. Although an increased motor unit recruitment has been reported when BFR is applied, as shown by greater electromyography levels [50], it is plausible that a minimal recruitment intensity is still necessary to elicit clinically meaningful adaptive responses. Abe and colleagues [44] suggested that the minimum intensity required for muscle hypertrophy in muscles subjected to BFR is approximately 10% of an MVIC [51]. Thus, it is plausible to speculate that strength gains and muscle thickness increases associated with BFR walking training depend on a minimum muscle activation threshold. This mechanistic understanding may be particularly relevant in early rehabilitation settings, such as post-ACL reconstruction, where voluntary activation is reduced due to arthrogenic muscle inhibition – a condition that can persist for up to two years post-surgery. In such cases, achieving even modest levels of activation through low-load BFR walking may suffice to promote beneficial neuromuscular adaptations [2,4].

Interestingly, the knee extensor and flexor muscles of the control limb (i.e., the side not subjected to BFR) also exhibited gains in strength following completion of the training protocol, but to a lesser extent (14.9% vs. 9.0%, respectively). It is essential to emphasize that the significant interaction indicates that the weaker limb experienced a more pronounced improvement in strength than the control limb. This highlights the intervention’s differential impact on the targeted (weaker) limb, which may have important practical implications for addressing inter-limb strength asymmetries – a common concern in rehabilitation settings, such as after ACL reconstruction. Strength improvements may result from distinct physiological mechanisms, involving neural adaptations and muscle hypertrophy. Neural mechanisms, including motor unit recruitment, firing rate, and interhemispheric facilitation, tend to dominate in the early phases of training and are particularly relevant in low-load conditions [44,52–54]. These neural adaptations are also responsible for the phenomenon of cross-education, in which training in one limb leads to strength gains in the contralateral, non-trained limb. In our study, the control limb (not exposed to BFR) demonstrated moderate strength gains, which may be partially explained by cross-education effects [44,52–54]. This is particularly relevant in unilateral rehabilitation protocols, where contralateral training may serve as an adjunct to preserve or improve strength. However, given that no direct neural or morphological assessments were performed, we cannot definitively distinguish the relative contribution of neural versus hypertrophic mechanisms in the observed adaptations. This limitation should be taken into account when interpreting the findings.

Although the BFR was applied to the thigh segment of the weaker limb (at the gluteal fold), significant strength gains were also observed in the muscles of the shank segment (i.e., the ankle muscles). Therefore, the ankle plantarflexors of occluded limb – although not the primary target of the intervention – showed a pronounced increase in strength (51.6%). Interestingly, this finding may have particular clinical relevance in patients undergoing ACL reconstruction, as persistent strength deficits are commonly observed not only in the thigh muscles but also in the muscles of the lower leg, especially the plantarflexors [21]. These deficits are frequently overlooked despite their known contribution to gait alterations and functional performance [21]. In this context, BFR-walking may represent a promising strategy to induce adaptations in both proximal and distal musculature, without requiring high mechanical loads, which is particularly advantageous in the early stages of rehabilitation.

Abe and colleagues reported that muscles surrounding the site of BFR application may undergo collateral hypertrophic adaptations and strength gains [51]. For instance, BFR applied at the proximal thigh can induce changes in adjacent muscles such as the gluteus, while BFR applied below the axilla may affect muscles like the pectoralis major. Therefore, muscular adaptations can occur both proximal and distal to the occlusion site, depending on the location of BFR application [51]. Bowman and colleagues [55] demonstrated BFR training, caused significant effects on proximal and distal muscles, particularly for the muscles where the tourniquet was applied (upper thigh). In the current study, the dorsiflexor muscles of the weaker limb showed only modest strength gains (~5%), which further reinforces the idea that the benefits of BFR may be linked to a minimum activation threshold, rather than simply being due to close to the vicinity of the tourniquet. It is known that during walking the gastrocnemius medialis activation is greater than tibialis anterior activation during most phase of gait [56–58], which would support this this contention.

The disparity between the results of the present study and previous studies using BFR walking training may be attributed to several factors, including the progressive walking training load and the relatively large BFR pressure (90%). Importantly, the present protocol adhered to the recommended 20 minutes maximum BFR, with sustained cuff inflation throughout the entire walking session. This differs from many BFR protocols that adopted intermittent approaches – alternating between inflation and deflation to allow blood flow to return. While this alternation inflation/deflation strategy may help manage discomfort, it could also reduce metabolic stress and limit adaptations, particularly during low-intensity training [59]. In contrast, the continuous occlusion may have enhanced the physiological stimulus, without compromising tolerability or safety. Further research is needed to examine the impact of BFR intermittency.

The hybrid training protocol implemented in this study, which included one in-person and two home-based sessions per week, demonstrated a high adherence rate, highlighting a positive aspect of the intervention. While using BFR walking training adds complexity to the intervention, due to safety concerns associated with the home-based sessions, adherence to the protocol was very high (~90%). This highlights that remote training can be successfully used for rehabilitation. The 90% adherence rate is higher than some of the adherence rates reported in fully supervised in-person training protocols [60–62]. Despite the potential discomfort associated with using BFR due to reduced circulation and metabolite accumulation, participants reported only light discomfort across the 12 weeks (mean = 3.4). This was in spite of the progressive nature of the training protocol, which suggests participants become acclimatized to the training regimen. Additionally, a significant increase in muscle strength was achieved through exercises perceived as very light to light in intensity (mean = 10.5), which reflects and confirms the effectiveness of the BFR walking training. No adverse or unexpected symptoms occurred, underscoring the protocol’s safety.

To better interpret the magnitude and clinical relevance of our findings, the Minimal Detectable Change (MDC) for each outcome was calculated. Notably, strength gains in the weaker limb exceeded the MDC for the plantarflexors and dorsiflexors, indicating that these changes were not only statistically significant but also large enough to surpass measurement error, and therefore can be considered clinically meaningful. For knee extensors and flexors, although statistically significant improvements were observed, the changes did not exceed the MDC threshold, highlighting the need for cautions interpretation regarding their clinical relevance. Regarding muscle thickness, changes in the GM and ST muscles exceeded the MDC in both limbs, while changes in the TA and VL only reached clinically meaningfulness in the weaker limb. Collectively, the findings indicate that the 12-week BFR walking protocol elicited meaningful improvements in the weaker limb. Strength gains in the plantarflexors and dorsiflexors exceeded the MDC, demonstrating changes beyond measurement error and supporting clinical relevance. Interlimb asymmetries observed at baseline were markedly reduced following the intervention. Specifically, ankle muscles showed restoration of strength symmetry, with plantarflexor and dorsiflexor differences decreasing from −23.9% and −10% at baseline to non-significant post-intervention values (p > 0.05). Knee extensors also exhibited near-complete recovery, with the strength difference decreasing from 26% at baseline to 3% post-intervention. In contrast, knee flexor strength showed a slight increase in asymmetry from 6.7% at baseline to 8.7% after the intervention (p = 0.024), indicating a relatively modest residual deficit. Although these absolute differences should be interpreted cautiously, the overall pattern underscores that the intervention was associated with reduced strength asymmetries as a result of clinically relevant adaptations in the targeted limb.

### 4.1. Study limitations

The present study has some limitations that must be acknowledged. The first limitation is the variation in time post-surgery (6–18 months) participants entered the study with, which may have led to differing responses across participants. Only participants with a persistent inter-limb asymmetry of at least 10% in knee extensor strength were recruited, which ensured there was a strength deficit in the weaker limb in all participant despite the time differences since surgery. Another limitation was that specific information regarding the surgical technique or graft type (e.g., patellar, hamstring, or quadriceps tendon) was not systematically collected. This was due to the fact that most participants were unable to recall the details of their surgery. While this information could be relevant – particularly in understanding potential neuromuscular inhibition patterns in the quadriceps or hamstrings – it was inconsistently available and thus was not part of the analysis.

The fact that physical activity and sedentary behavior were assessed through self-reported questionnaires may also be considered a limitation. While these tools are commonly used in rehabilitation research due to their practicality, they are subject to recall bias and limited sensitivity – particularly in detecting light-intensity activities, short bouts of movement, and day-to-day variability – which may affect the accuracy of these measurements. However, it is important to note that physical activity was included primarily for participant characterization and not as a primary outcome of the study, and thus this limitation was deemed acceptable. Additionally, caloric intake was not recorded during the study. Although participants were instructed to maintain their usual diet, we cannot rule out the possibility that changes in energy intake may have influenced body mass outcomes.

Muscle thickness measures were used as a proxy for muscle hypertrophy, which thus can be considered a limitation, because thickness only provides a unidimensional view of a single transverse site of the muscle and does not account for changes in cross-sectional area or muscle volume.

The lack of a no-intervention control group is also a limitation. This means that we cannot be completely certain that the benefits observed were solely a result of the BFR therapy or were simply due to the walking training. Future randomized controlled trial studies are needed to fully assess the benefits of BFR walking therapy. Nonetheless, the within-subject design offers some important advantages over between-subject comparisons. It minimizes confounding effects related to having two groups with differing lifestyle and baseline characteristics, which can be a problem in control group trials. This benefit may be particularly relevant in rehabilitation contexts, where individual histories and capacity for physiological adaptation (i.e., adaptive margins) can vary widely. In the current study, the operated limb was designated the weaker limb (WL) and subjected to blood flow restriction (BFR), while the unoperated (i.e., stronger contralateral limb) served as the control limb (CL). Using the contralateral segment as a control ensured comparable exposure to daily activities, which is difficult to achieve in a study with two independent groups. However, the absence of a no-intervention control group does limit the internal validity of the findings. Although adherence procedures were implemented, including the option for participants to provide photo or video evidence of training sessions, this was not mandatory. Therefore, intervention fidelity relied partially on self-reported data, which may introduce bias and thus should also be considered a limitation. Future randomized controlled trials with parallel groups and adequate control conditions are warranted to confirm and expand upon these results.

## 5. Conclusions

This study represents an advancement in ACL rehabilitation, as it is the first to implement a walking protocol combined with BFR using a within-subject experimental design. In addition, it is the first study to indicate that a walking protocol combined with BFR may be effective in producing marked strength improvements in the operated limb (the weaker limb, where the BFR was applied) compared to the control limb (the stronger limb, which had no BFR applied). Furthermore, the hybrid training protocol, which combined in-person and home-based sessions, appeared to preserve adherence while also maintaining safety aspects. Early rehabilitation protocols are crucial for muscle recovery and protecting the healing ligament and graft site. Low training loads are preferred over traditional ‘over-load’ stimuli, which are usually applied to elicit strength and hypertrophic responses. Low-intensity BFR walking training may provide a means for patients to strengthen their muscles without placing excessive stress on the healing tissues, thereby promoting a safer recovery process. This method may represent a promising alternative to traditional high-load exercises during the critical early stages of rehabilitation. Nonetheless, these findings should be interpreted with caution, as they derive from a sample of young adults with persistent strength asymmetries and varying postoperative timelines. Further research is needed to determine their applicability to broader clinical populations.

### Highlights

- BFR walking was associated with improvements in ankle and knee strength after ACL reconstruction;
- The weaker limb showed greater strength gains than the control limb;
- Extensor muscles showed greater strength gains than flexor muscles;
- Gastrocnemius thickness increased on both sides, regardless of the BFR intervention;
- High adherence and low discomfort support feasibility of hybrid BFR rehabilitation.

## Supporting information

S1 File Study Protocol. Detailed description of the theoretical study design and methodology.
(PDF)

S2 File Study Dataset. Raw data used for the analyses reported in the manuscript.
(XLSX)
