# Supplementary material for: Walking with blood flow restriction on lower limb muscles post-ACL reconstruction: A within-subject trial
Source: PLoS One. 2025 Oct 8;20(10):e0333200. doi: 10.1371/journal.pone.0333200 (PMC12507242; doi:10.1371/journal.pone.0333200)
Supplement: S1 File — Detailed description of the theoretical study design and methodology. (PDF) [file pone.0333200.s001.pdf]

Title: Effects of a Walking Program Combined with Partial Blood Flow Restriction on Muscular and Functional Aspects in Individuals with Lower Limb Muscle Strength Asymmetry

Running title: Walking with Partial Blood Flow Restriction and Muscle Strength Asymmetry

Ethics approval: 07th August, 2024

Sponsor: Federal University of Paraná.

SIGNATURE PAGE

The undersigned confirm that the following protocol has been agreed and accepted and that the Chief Investigator agrees to conduct the trial in compliance with the approved protocol and will adhere to the principles outlined in the Brazilian Clinical Trials Registry (Clinical Trials). I agree to ensure that the confidential information contained in this document will not be used for any other purpose other than the evaluation or conduct of the clinical investigation without the prior written consent of the Sponsor. I also confirm that I will make the findings of the trial publically available through publication or other dissemination tools without any unnecessary delay and that an honest accurate and transparent account of the trial will be given; and that any discrepancies and serious breaches of REBEC from the trial as planned in this protocol will be explained.

Chief Investigator:

Dr. André Rodacki

Date: 03/06/2025

1.0 Trial Summary

**Trial title**	Effects of a Walking Program Combined with Partial Blood Flow Restriction on Muscular and Functional Aspects in Individuals with Lower Limb Muscle Strength Asymmetry	
**Short Title**	Walking with Partial Blood Flow Restriction and Muscle Strength Asymmetry	
**Trial Design**	Non-randomized clinical trial with a within-subject design	
**Trial Participants**	Participants confirmed with ACL reconstruction	
**Planned Sample Size**	27	
**Treatment**	Walking with blood flow restriction (training program)	
**Follow-up**	Assessment pre- and post- intervention (12 weeks)	
**Planned Trial Period**		
	**Objectives**	**Outcome Measures**
**Primary**	a) The training induces changes in muscle strength	a) Muscle Strength
**Secondary**	a) The training induces changes in anthropometric data<br>b) The training induces changes in physical activity level and sedentary behavior<br>c) The training induces changes in muscle thickness<br>d) The training induces changes in gait analysis	a) Anthropometric Data<br>b) Physical Activity Level and Sedentary Behavior<br>c) Muscle Thickness<br>d) Gait Analysis

2.0 Overview of study

2.1 Aim

To verify whether different types of muscle strength asymmetries in the lower limbs (i.e., primary and secondary) can be minimized through a walking training program combined with partial blood flow restriction.

2.1.1 Hypothesis

i) Individuals with a history of anterior cruciate ligament (ACL) injury will exhibit greater muscle strength asymmetry between limbs compared to healthy individuals, identified through the knee extensor musculature;

ii) The exercised limb will demonstrate a significant increase in muscle strength around the ankle and knee joints compared to the control limb, and consequently, will show a reduction in muscle strength asymmetry between lower limbs, in both healthy adults and those with ACL injury;

iii) The exercised limb will demonstrate a significant increase in muscle volume around the ankle and knee joints compared to the control limb, in both healthy adults and those with ACL injury;

iv) The walking training program combined with partial blood flow restriction will have a positive impact on gait performance at different walking speeds, in both healthy adults and those with ACL injury.

2.2 Background

Muscle strength asymmetry is defined as the lack of equality between limbs or muscle groups (PARKINSON et al., 2021) and has become a common subject of investigation in recent years due to its effects on injury occurrence and performance reduction (BISHOP; TURNER; READ, 2018). Bilateral movements depend on symmetry and balance between body sides and form the foundation of many simple and complex actions (ATKINS et al., 2016). Conceptually, asymmetries can be classified as primary (i.e., not associated with injuries and/or diseases) or secondary (i.e., caused by the development of primary injuries and/or diseases).

Symmetry is related to aspects of physical fitness (PAWLOWSKI et al., 2018), which are susceptible to environmental factors such as physical inactivity and sedentary behavior. These factors are recognized as public health issues (OZEMEK; LAVIE; ROGNMO, 2019; PRATT et al., 2020), which result in physical and functional decline and contribute to changes and the

development of asymmetries. Additionally, lateral preference (left or right), which refers to the preferential use of one side of the body in voluntary motor acts, may lead to the development of inter-limb asymmetries due to the continuous and excessive use of one body side (e.g., tennis player's dominant arm) (ZUMSTEIN; CENTNER; RITZMANN, 2022).

Muscle strength asymmetry can negatively affect an individual's physical capacity due to the potential onset of injuries and performance reduction, especially in sports skills (ATKINS et al., 2016; BISHOP; TURNER; READ, 2018). In asymmetric limbs, the weaker limb will produce less force and reach its tolerance threshold earlier compared to the stronger limb during high-intensity repeated actions (FORT-VANMEERHAEGHE et al., 2020). Meanwhile, the stronger limb may endure a greater amount of excessive stress due to increased reliance and higher load absorption (GUAN et al., 2022). Therefore, the risk of injury may increase, as individuals with greater inter-limb strength asymmetry have lower ability to perform unilateral technical tasks with higher quality (FORT-VANMEERHAEGHE et al., 2020). Thus, maintaining balance between the right and left sides is of great importance, as individuals with deficits greater than 15% show a higher incidence of injuries compared to groups with asymmetries below this threshold (ATKINS et al., 2016; BISHOP; TURNER; READ, 2018). However, it is important to note that differences of approximately 10% between body sides may also be considered clinically significant (BISHOP et al., 2018; SARABON et al., 2020).

Inter-limb asymmetries can result in unequal force absorption or loss of frontal plane stability, both of which are important for withstanding impact forces (GUAN et al., 2022). Increased inter-limb asymmetry during walking, combined with muscle strength deficits, are known risk factors for injuries, especially during multi-joint movements of the lower body. One of the most common injuries with high prevalence is anterior cruciate ligament (ACL) injury, which is the most frequently injured knee ligament (HUGHES et al., 2018; ITHURBURN et al., 2015; JORDAN; AAGAARD; HERZOG, 2015).

Following an ACL injury, the main goal is to restore ligament stability and allow the individual's functional return. Therefore, ACL reconstruction surgery (ACLR) is usually performed (HUGHES et al., 2018; ITHURBURN et al., 2015).

One of the main consequences of ACL injury and subsequent surgery is thigh muscle atrophy, particularly of the quadriceps (HUGHES et al., 2018). Muscle weakness tends to occur

bilaterally due to conditions related to rest, but it is significantly more pronounced in the injured limb, contributing to the development of secondary asymmetry. Quadriceps muscle weakness is debilitating and can contribute to overall dysfunction while increasing the risk of re-injury and the early onset of osteoarthritis. This asymmetry can be observed primarily within the first 12 weeks after surgery but may persist for more than 2 years post-operation (HUGHES et al., 2018). Furthermore, it negatively affects physical function, quality of life, and the recovery process of individuals (HUGHES et al., 2018).

Asymmetry may be present even before the injury and become worse during the ACL injury or may arise after ACL reconstruction (ACLR). Therefore, symmetrical thigh strength, particularly of the quadriceps, should be a key goal of rehabilitation, since greater inter-limb asymmetry after ACLR is associated with self-reported dissatisfaction, reduced functional performance, and altered lower limb mechanics during gait (PALMIERI-SMITH; LEPLEY, 2015).

Furthermore, gait asymmetries after ACLR are evident as early as 6 months post-surgery and may persist for many years. Asymmetrical movements of the lower limbs may contribute to the development of osteoarthritis and increase the risk of re-injury (TAJDINI et al., 2021). Previous studies show that athletes with biomechanical asymmetries at the time of return to sport are three times more likely to suffer a re-injury within a year compared to those without asymmetries (HEWETT et al., 2005). Asymmetrical load distribution in the lower limbs may also alter chondrocyte synthesis and catabolic activity, leading to structural damage of the articular cartilage and accelerating the development of knee osteoarthritis (CARTER et al., 2004; CHMIELEWSKI, 2011). The reduction in quadriceps muscle strength, combined with psychological factors such as fear of movement, fear of reinjury, lack of confidence, and self-efficacy, may have a direct relationship with gait asymmetry (HUGHES et al., 2018; ZARZYCKI et al., 2018).

Therefore, due to the need to restore individuals to normal function with a low risk of recurrence, it is crucial to maximize thigh muscle strength and reduce inter-limb asymmetry to preserve knee joint health and functional capacity (PALMIERI-SMITH; LEPLEY, 2015). The development of muscle strength usually requires repeated recruitment of high-threshold motor units to induce tissue tension or the necessary physiological response for adaptation. For this to occur, training typically requires loads of 65% to 70% of one-repetition maximum (1RM).

However, there are several contraindications to this type of exercise, as this intensity may produce stress loads that the newly reconstructed ACL cannot tolerate (HUGHES et al., 2018).

Additionally, a significant proportion of individuals present alterations after ACL reconstruction (ACLR) and throughout the recovery process. A systematic review and meta-analysis reported that among 5,770 individuals, only 60% returned to pre-injury activity levels, 45% returned to competitive sports (ARDERN et al., 2016), and between 6% and 29.5% suffered a re-injury within 24 months (PATERNO et al., 2014; SALMON et al., 2005; WRIGHT et al., 2007).

Given this, professionals face the challenge of finding alternative tools to ensure successful ACL rehabilitation and reduce recovery time. It is necessary to increase muscle strength and size, while reducing inter-limb asymmetry, without imposing undesirable stress loads on the joint. Therefore, blood flow restriction (BFR) training has been proposed as a tool for early post-ACL surgery rehabilitation due to its low load nature and hypertrophic capacity (HUGHES et al., 2018). BFR is commonly performed using pneumatic tourniquets, inflatable cuffs, or elastic bands, and involves partial and total restriction of arterial and venous blood flow, respectively (KILGAS et al., 2019). Furthermore, it can be used progressively at all stages of rehabilitation, from early postoperative phases to return to heavy load exercise and pre-injury activity levels (HUGHES et al., 2018).

The nature of BFR allows individuals with contraindications for heavy load exercises to benefit from training programs that promote hypertrophy and strength gains (KILGAS et al., 2019). However, despite positive results observed in different populations (e.g., athletes to severely ill individuals) from combining low-load resistance training (20–50% 1RM) with BFR (LIXANDRÃO et al., 2018), and more specifically for ACL rehabilitation (ÁLVAREZ et al., 2021; CHARLES et al., 2020; COLAPIETRO et al., 2022; KOC et al., 2022), there are various methods of applying BFR that can be explored and have not been discussed in detail.

Among these, walking with BFR stands out, where participants walk with an inflated cuff around the thigh (unilaterally or bilaterally). Walking with BFR has been shown to increase muscle strength and size, and improve several aspects of physical function in different populations (ABE et al., 2010; ABE, KEARNS, SATO, 2006; CLARKSON, CONWAY, WARMINGTON, 2017; HUGHES et al., 2017; KARGARAN et al., 2021; OZAKI, H. et al.,

2011; OZAKI, HAYO et al., 2011; VECHIN et al., 2015). Abe and colleagues (2010) reported increases of 5.8% in cross-sectional area and 10.7% in quadriceps muscle mass after six weeks of walking with BFR in older adults (ABE et al., 2010). Despite the beneficial effects of walking with BFR (ABE, KEARNS, SATO, 2006; BEEKLEY, SATO, ABE, 2005; CLARKSON, CONWAY, WARMINGTON, 2017; FARAS et al., 2019; KILGAS et al., 2019; OZAKI, H. et al., 2011; OZAKI, HAYAO et al., 2011), its effects on inter-limb asymmetries in individuals with ACL injury remain unclear. The potential to produce more pronounced hypertrophic responses in a single segment is interesting, especially to reduce discrepancies between body halves. It is well known that hypertrophic adaptations in young and physically active adults are more difficult than in sedentary older adults, given that adaptability margins are considerably different (HORTOBÁGYI et al., 2003). Therefore, it is unknown whether adults with inter-limb asymmetries in the lower limbs will present significant responses when subjected to walking with BFR. Given this, the present study aims to verify whether different types of lower limb muscle strength asymmetries (i.e., primary and secondary) can be minimized through a walking training program combined with partial blood flow restriction.

2.3 Rationale

Muscle strength asymmetry is defined as the lack of equality in muscle mass or in the ability to produce force between limbs or muscle groups (Parkinson et al., 2021). Asymmetries in force production capacity are problematic and have been investigated due to their negative impact on physical performance—particularly in sports—and their association with increased injury incidence (Atkins et al., 2016; Bishop, Turner, & Read, 2018). Values of approximately 10% are considered clinically significant; however, discrepancies as small as 5% have also been linked to reduced performance (Bishop et al., 2018; Sarabon et al., 2020). Therefore, maintaining symmetrical balance between limbs is important, and effective strategies should be implemented to reduce such imbalances (Kyritsis et al., 2016; Rohman, Steubs, & Tompkins, 2015).

Training programs incorporating a variety of exercises have been developed as an effective strategy to combat physical inactivity and sedentary behavior (Mok et al., 2020), both of which are recognized as “global pandemics” (Ozemek, Lavie, & Rognmo, 2019; Pratt et al., 2020). Such programs lead to improvements in physical and functional capacities, which can help prevent the development of physical alterations (e.g., asymmetries). Resistance training programs with high loads (60–85% of one-repetition maximum [1RM]) are commonly used to

promote muscle strength and hypertrophy (Giles et al., 2017). However, these programs tend to elicit high perceptual responses, including increased perceived exertion and pain, which can pose barriers to adherence, particularly when pre-existing injuries are present and may be exacerbated by the nature of traditional resistance training. Therefore, low-intensity training protocols that generate comparable outcomes are particularly appealing (Fallon et al., 2022; Faras et al., 2019; Mok et al., 2020).

An effective alternative is blood flow restriction (BFR) training, which involves the partial occlusion of blood flow to muscles during exercise (Kilgas et al., 2019). BFR training at 20–30% of 1RM can induce hypertrophy, enhance strength, and improve both physical and functional capacity, including maximal aerobic capacity (Giles et al., 2017; Mok et al., 2020). These adaptations have been observed not only with resistance exercises, but also with multi-joint movements such as walking (Mok et al., 2020).

Walking with BFR is performed using an inflatable cuff placed around the thigh (unilaterally or bilaterally), and it has been shown to increase muscle size and strength (Abe et al., 2010; Abe, Kearns, & Sato, 2006; Clarkson, Conway, & Warmington, 2017; Hughes et al., 2017; Kargaran et al., 2021; Ozaki, H. et al., 2011; Ozaki, Hayao et al., 2011; Vechin et al., 2015). Abe et al. (2010) reported increases of 5.8% in cross-sectional area and 10.7% in quadriceps muscle mass after six weeks of BFR walking training in older adults. Despite the documented benefits of walking with BFR (Abe, Kearns, & Sato, 2006; Beekley, Sato, & Abe, 2005; Clarkson, Conway, & Warmington, 2017; Faras et al., 2019; Kilgas et al., 2019; Ozaki, H. et al., 2011; Ozaki, Hayao et al., 2011), its effects on muscle strength asymmetry in individuals remain unclear.

It is known that hypertrophic adaptations in physically active adults are more difficult to achieve than in sedentary older adults, given that adaptive potential is considerably different between these populations (Hortobágyi et al., 2003). Thus, it remains uncertain whether adults with lower-limb inter-limb asymmetries will demonstrate significant responses to walking with BFR. In light of this, the present study aims to investigate whether different types of muscle strength asymmetry in the lower limbs (i.e., primary and secondary) can be reduced through a walking training program combined with partial blood flow restriction.

2.4 Assessment and management of risk

Data collection procedures may pose risks of embarrassment and fatigue to participants due to biological and social individuality. During or shortly after the assessments and training sessions, participants may experience mild muscle soreness resulting from the movements performed. However, such discomforts are transient and have been reported as less intense compared to high-load resistance training, due to the low intensity of the exercises.

One strategy to prevent adverse effects is the inclusion of rigorous eligibility criteria, as described in section 5.3, which are directly related to the practice of exercise with blood flow restriction (BFR) (Fallon et al., 2022). Evidence suggests that BFR training, when conducted in a controlled environment by trained and experienced professionals, offers a safe training alternative for most individuals, regardless of age or fitness level (Loenneke et al., 2011).

Moreover, the research team has prior experience with this type of training, as demonstrated by related publications that confirm extensive knowledge of the techniques and potential risks associated with the protocols applied in the study:

1. Skiba, G. H.; Andrade, S. L. F.; Rodacki, A.L.F. Effects of functional electrostimulation combined with blood flow restriction in affected muscles by spinal cord injury. Neurological Sciences, v. 42, p. 1–10, 2022.
2. Andrade, S. L. F.; Skiba, G. H.; Krueger, E.; Rodacki, A. L. F. Effects of Electrostimulation with Blood Flow Restriction on Muscle Thickness and Strength of the Soleus. Journal of Exercise Physiology Online, v. 19, p. 59–69, 2016.

Mild muscle soreness may occur following the pre- and post-intervention assessments due to the maximal isometric muscle strength test (Biodex). It is important to emphasize that the walking training program combined with blood flow restriction (BFR) may cause discomfort only during the session. After releasing the BFR and restoring blood circulation, the discomfort is expected to cease immediately. Resistance training causes muscle soreness due to its mechanism of promoting muscle strength and hypertrophy through microtrauma to muscle fibers. In contrast, BFR exercises promote positive muscular adaptations through reduced blood flow to the target area, leading to localized metabolic stress.

The research will be conducted in a private setting, on an individual basis, by a previously trained researcher. Participants may discontinue the assessments at any time. Tests will be

organized in a circuit format to minimize localized fatigue, pain, and performance impairment. A recovery interval of approximately two minutes will be allowed between tests, and participants may stop the procedures at any point. If any signs or symptoms such as fatigue, pain, or significant discomfort arise, the test will be interrupted.

Only participants with medical clearance (an eligibility criterion) provided by the physician associated with the study will be allowed to participate. The physician will also provide free medical support to any participant who experiences negative effects. All of the above measures are intended to minimize the risk of adverse events during the study period. Nevertheless, if any occur, appropriate actions will be taken to ensure immediate care and treatment for the participant.

2.5 Programme and Methodology

2.5.1 Research design

Non-randomized clinical trial with a within-subject design.

2.5.2 Participant identification and recruitment

This study will be conducted in Curitiba, a city with a population of 1,894,000 inhabitants, according to estimates from the Institute for Research and Urban Planning of Curitiba (Instituto de Pesquisa e Planejamento Urbano, 2017). The sample size calculation will be performed using G*Power 3.1 software (University of Düsseldorf, Düsseldorf, Germany). The effect size (0.4) was derived from the study conducted by Lamberti et al. (2020), which investigated the effects of a walking protocol combined with partial blood flow restriction on walking speed. The following parameters were considered for the calculation: (i) F test (ANOVA); (ii) 95% confidence level; (iii) maximum sampling error of 5%; (iv) statistical power of 90%; (v) number of groups = 2; and (vi) number of measurements = 2 (pre- and post-intervention assessments). Accordingly, the minimum required sample size for this study will be 52 participants. An additional 5% will be added to account for potential dropouts or data loss, resulting in a final required sample size of 54 individuals, divided into two groups of 27 participants (intervention segment vs. control segment).

2.5.2.1 Inclusion/exclusion criteria

The eligibility criteria for inclusion in the training program will be as follows: (i) individuals aged between 18 and 59 years; (ii) absence of conditions that may hinder the completion of

questionnaires, understanding of instructions, or performance of tests (e.g., severe vestibular deficits and mobility impairments); (iii) no cognitive impairment that could interfere with questionnaire completion, comprehension of instructions, or execution of the testing procedures; (iv) a strength asymmetry of ≥10% between the lower limbs in the knee extensor musculature (quadriceps); (v) medical clearance to participate in the training program, issued by the physician associated with the study (Dr. Francisco Wekerlin Morozowski); (vi) systolic blood pressure ≤140 mmHg or diastolic blood pressure ≤90 mmHg; and (vii) absence of more than one thromboembolism risk factor, which includes: (1) body mass index ≥30 kg/m², (2) diagnosis of chronic inflammatory disease, (3) history of fractures in the pelvis, hip, or femur, (4) major surgeries within the past six months, (5) diagnosis of varicose veins within the past six months, (6) family history of deep vein thrombosis or pulmonary embolism, (7) use of oral contraceptives, or (8) tobacco use (Fallon et al., 2022). Participants who do not meet the eligibility criteria will be excluded from the study, as well as those who do not sign the informed consent form or fail to complete at least 75% of the proposed training sessions, if selected for the intervention. All criteria will be assessed during an initial screening conducted by the researchers and the study physician.

2.5.3 Protocol and intervention

A familiarization session will be conducted prior to the beginning of the training program with the purpose of introducing the research project, demonstrating the procedures related to blood flow restriction (BFR), and addressing any questions the participants may have. Additionally, during this initial meeting, the physician responsible for the study will conduct an individual assessment of all participants and provide medical clearance for participation in the training protocol.

Participants will undergo training three times per week over a 12-week period, totaling 36 sessions. Researchers will develop a structured training protocol to maximize adherence, in which one session per week will be conducted in person (12 sessions), and two sessions per week will be performed at home without researcher supervision (24 sessions). Training sessions will be consistently scheduled on the same days each week (Tuesdays [in-person], Thursdays and Saturdays [home-based]). Participants will be instructed to attend at least 75% of the proposed sessions, that is, a minimum of 27 sessions. If one or more sessions are missed, rescheduling will be encouraged and coordinated based on participant and researcher availability.

Throughout all in-person sessions, the lead researcher, accompanied by at least two assistant researchers, will supervise all procedures and provide necessary guidance, emphasizing protocol consistency to ensure participant safety (e.g., cuff positioning, accurate BFR application, and training intensity). Participants will be divided into subgroups and assigned to different time slots to allow researchers to properly monitor the sessions and provide individualized attention. Heart rate (HR) and blood pressure (BP) will be measured before and after each training session using a digital sphygmomanometer. Furthermore, the rating of perceived exertion (RPE, Borg Scale) and discomfort scale (VAS, Visual Analog Scale) will be administered five minutes after the conclusion of each session. An attendance sheet and adverse event report will be completed during each session to assess adherence and identify any potential adverse effects. For the home-based sessions, participants will follow the same procedures as those used during in-person training, and will be instructed to maintain a log with session details (i.e., blood pressure, heart rate, and any occurrences).

An intra-subject design will be adopted in this study, in which the weaker limb will undergo the training and will be referred to as the "intervention limb" (IL), whereas the stronger limb will serve as the "control limb" (CL). This methodology offers substantial advantages, such as reducing inter-participant variability, greater control over daily life variability, more robust and consistent analysis, and increased statistical power and sensitivity, particularly in longitudinal designs (MONTOYA, 2023). Although systemic effects of BFR are acknowledged, the primary focus of this study will be to assess the local impact of the training on the intervention limb.

Initially, HR and BP will be measured after five minutes of rest. Subsequently, a cuff will be placed around the thigh of the IL, at 25% of the distance between the greater trochanter and the lateral femoral condyle, to induce blood flow restriction during the training protocol (FARAS et al., 2019). Participants will then shift their body weight onto the leg, and the cuff will be inflated to 90% of the individual's blood flow restriction pressure (BFRP), previously determined in the initial assessment using a vascular Doppler. The cuff will remain inflated and secured throughout the entire training session (i.e., walking). Cuff pressure will be monitored and adjusted if necessary (i.e., in case of pressure drop). Upon completion of the session, the cuff will be slowly deflated and removed, and participants will be instructed to walk for at least two minutes to facilitate blood circulation restoration. Walking will be performed in small

groups, in a flat and unobstructed environment, and cadence will be standardized using a free metronome application (Cifra Club®).

The blood flow restriction pressure (BFRP, mmHg) will be individually determined using a manual sphygmomanometer (Premium®, model BR 20D; 18 cm width) and a vascular Doppler device (Martec®, model DV600). Participants will remain in the supine position while the sphygmomanometer cuff is placed in the inguinal region (i.e., near the gluteal fold) of the asymmetric limb and gradually inflated at a rate of 2 mmHg per second until the auscultatory pulse of the tibial artery is no longer detected (LAURENTINO et al., 2008). The lowest pressure at which the pulse disappears will be recorded as the individual's arterial occlusion pressure (FALLON et al., 2022). For BFR training sessions, a pressure equivalent to 90% of the BFRP will be applied in order to maximize the recruitment of fast-twitch muscle fibers and promote muscular adaptations (FATELA et al., 2016; HUGHES et al., 2017; LIXANDRÃO et al., 2015).

The training protocol will be divided into six progressive phases in which time and cadence will be gradually adjusted to increase training intensity. The duration, speed, and distance of each protocol phase will be presented in Table 1.

Table 1. Blood flow restricted training protocol parameters across 12 weeks (36 sessions).

Phases	Weeks	Time	Cadence	Speed	Distance	Duration
**Phase 1**	Week 1<br>Week 2	12 min	110 steps.min^{-1}	1.25 m.s^{-1}	900m	2 weeks
**Phase 2**	Week 3	14 min	110 steps.min^{-1}	1.30 m.s^{-1}	1100 m	1 week
**Phase 3**	Week 4	16 min	110 steps.min^{-1}	1.36 m.s^{-1}	1300 m	1 week
**Phase 4**	Week 5	18 min	115 steps.min^{-1}	1.47 m.s^{-1}	1600 m	1 week
**Phase 5**	Week 6<br>Week 7<br>Week 8	20 min	115 steps.min^{-1}	1.91 m.s^{-1}	2300 m	3 weeks
**Phase 6**	Week 9<br>Week 10<br>Week 11<br>Week 12	20 min	120 steps.min^{-1}	2.00 m.s^{-1}	2400 m	4 weeks

The rating of perceived exertion (RPE) will be assessed using the Borg Scale, which ranges from 6 to 20 points, where 6 indicates "no exertion" and 20 indicates "maximum exertion and inability to continue" (BORG, 1998). Additionally, the Visual Analog Scale (VAS) will be applied to monitor pain resulting from the training session. The VAS is a 10-point scale with descriptors at 0 (no pain), 5 (moderate pain), and 10 (worst imaginable pain) (BREIVIK et al.,

2008). The VAS will be established as a reliable and valid measure of pain intensity, especially for assessing acute pain induced by BFR (BROWN et al., 2018; SHARMA et al., 2014). Both scales will be administered individually, five minutes after each training session, to avoid symptom exacerbation. Participants will receive verbal instructions regarding the use of these scales during the familiarization session and will be reminded at each training session.

2.5.4 Outcome measure and analysis

2.5.4.1 Sociodemographic and Clinical Data

Information regarding age, gender, anthropometric data, physical activity level, sedentary behavior, history of fractures and musculoskeletal injuries (within the past 12 months), and self-rated health status (poor, fair, good, or very good) will be collected through an individualized interview.

2.5.4.2 Anthropometric Profile

The participants' anthropometric profile will be determined based on the following measurements: height (cm), body mass (kg), and Body Mass Index (BMI, kg/m²). Height, measured in centimeters (cm), will be assessed using a portable stadiometer (WISO®) with a 1 mm scale. Participants will be instructed to stand barefoot in an anatomical position, with their head and trunk as upright as possible, head aligned parallel to the ground, and body weight evenly distributed on both feet. Once properly positioned, the stadiometer cursor will be placed at a 90º ankle in relation to the scale, touching the highest point of the head (Guedes & Guedes, 2006). Body mass, measured in kilograms (kg), will be assessed using a portable digital scale with a 0.1 kg precision and a maximum capacity of 150 kg. Participants will stand barefoot, wearing light clothing, facing forward with body weight evenly distributed on both feet (Guedes & Guedes, 2006). Based on these measurements, BMI will be calculated using the following formula: BMI = body mass (kg) / height $(m)^2$ (Eknoyan, 2008).

2.5.4.3 Physical Activity Level and Sedentary Behavior

The level of physical activity will be assessed using the Brazilian version of the Internation Physical Activity Questionnaire (IPAQ) (Matsudo et al., 2001), which consists of seven open-ended questions designed to estimate the time spent per week in various dimensions of physical activity (light, moderate, and vigorous intensities) and sedentary behavior (sitting, reclining, and/or lying down positions). Additionally, the total physical activity level (minutes/week) will

be used to classify participants as insufficiently active (<150 minutes/week), active (150-300 minutes/week), or very active (>300 minutes/week), according to the American College of Sports Medicine guidelines (Chodzko-Zajko et al., 2009). Sedentary behavior will be assessed through two questions from the IPAQ, considering the time spent (in hours) in routine sedentary activities on weekdays and weekends (Matsudo et al., 2001).

2.5.4.4 Muscle Strength

Lower limb muscle strength will be assessed using the Biodex Multi-Joint System dynamometer (Biodex Medical Systems Inc., Shirley, NY, USA), through the measurement of maximal voluntary isometric contraction (MVIC) of the ankle plantiflexors and dorsiflexors, and the knee extensors and flexors. Isometric strength measurements are highly relevant and considered the gold standard for monitoring training-related adaptations (Arhos et al., 2021; Smajla, Žitnik, & Šarabon, 2021). For the evaluation of ankle musculature, participants will be seated in the isokinetic dynamometer chair with the backrest reclined at a 70º angle. The backrest will be adjusted so that the sole of the foot is fully supported by the lever arm platform. The device's axis of rotation will be aligned with the lateral malleolus of the ankle (the anatomical axis of ankle rotation). The knee will be positioned at approximately 30º of flexion. Participants will be instructed to avoid compensatory movements, particularly those involving the hip. The limb being assessed will be weighed in a relaxed, neutral position (0º) to correct for the effect of gravity.

For the evaluation of knee musculature, participants will be seated in the dynamometer chair with the hip joint positioned at a 90º angle. The backrest will be adjusted until the popliteal fossa is in contact with the seat. The device's axis of rotation will be aligned with the lateral femoral epicondyle (the anatomical axis of knee rotation). The chair's backrest will remain at 90º, and the lever arm pad will be adjusted and fixed 2 cm above the lateral malleolus. Participants will be instructed not to perform plantarflexion or dorsiflexion during the test. The tested limb will be weighed in a relaxed position with the knee in semi-extension (45º) to correct for gravitational effects.

In both tests, participants will be stabilized using two thoracic straps, one pelvic strap, and Velcro straps over the metatarsal region of the evaluated foot (for ankle testing) and over the distal thigh (for knee testing). They will be instructed to hold the side handles of the chair to

prevent any change in body position during testing and to avoid assistance from other muscle groups.

After the participant is properly positioned, a warm-up and familiarization phase will be conducted, consisting of four MVIC repetitions for the ankle plantar flexors and dorsiflexors at 0º and for the knee extensors and flexors at 60º, with a 60-second interval between contractions (Andersen et al., 2012). One minute after the warm-up, participants will be instructed to perform three MVICs at 60º, each lasting 5 seconds, with 120-second intervals between repetitions, aiming to achieve maximal isometric contraction (Eriline et al., 2011). During all repetitions, participants will receive standardized verbal encouragement and visual feedback via the torque curve displayed on the dynamometer monitor to maximize performance (Dotan et al., 2013).

Peak torque (PT), expressed in Newton-meters (N·m), will be defined as the best performance among the three trials and calculated as the product of peak force (N) and the distance from the point of force application to the segment's center of rotation (m) (Bento et al., 2010). Isometric peak torque (N·m) will be normalized by total body mass (N·m/kg) (Aagaard et al., 2009). Finally, inter-limb strength asymmetry will be calculated using peak torque values of the knee extensors based on the following formula (Beato et al., 2021):

MSI = (Weaker limb / Stronger limb) x 100

2.5.4.5 Muscle Thickness

Participants who agree to take part in the training program will undergo muscle thickness (MT) assessments both before and after the intervention period. The analysis will be performed using B-mode ultrasound with a portable device (Konica Minolta®, Sonimage HS1, Version 1.10) equipped with a transducer measuring 5 cm in length and 2 cm in width, operating at a frequency of 11 MHz (4x2).

In adults, ultrasound has been described as a valid and reliable method for measuring muscle size, including muscle thickness, due to its accessibility, cost-effectiveness, and non-invasive nature. MT will be identified as a valid correlate of muscle volume and cross-sectional area when compared to magnetic resonance imaging in adult populations (ROCK et al., 2021), and will be estimated as the distance between the superficial and deep aponeuroses (GANDOLFI et

al., 2018; KARAGIANNIDIS, 2017). Participants will be instructed to avoid any lower limb exercise 48 hours before image acquisition.

Before the measurements, participants will remain in the supine position with a relaxed body and extended knees for at least 15 minutes to allow fluid redistribution (BERG; TEDNER; TESCH, 1993; TICINESI et al., 2018). A previously trained examiner will perform the ultrasound procedure, applying a thick layer of water-soluble transmission gel to the transducer to ensure acoustic contact without compressing the skin. The transducer will be positioned parallel to the muscle's longitudinal axis and perpendicular to the tissue surface. Scan depth will be fixed at 5 cm for all measurements and increased only when necessary, in participants with higher subcutaneous fat, to identify aponeuroses and capture an appropriate muscle area.

Measurements will be taken for the tibialis anterior (TA), medial gastrocnemius (MG), vastus lateralis (VL), and semitendinosus (ST) muscles:

- Tibialis anterior (TA): Participant in supine position with knees and hips in 0° of flexion, abduction, and rotation. The measurement point will be at 20% of the distance between the fibular head and medial malleolus (FRAIZ et al., 2020).
- Medial gastrocnemius (MG): Participant in prone position with extended knees and feet hanging off the examination table, with the ankle relaxed at approximately 115°. The measurement site will be at 30% of the distance between the popliteal fossa and the medial malleolus (MCCREESH; EGAN, 2011).
- Vastus lateralis (VL): Participant in supine position with knees and hips in 0° of flexion, abduction, and rotation. The site will be at 50% of the distance between the greater trochanter and the lateral border of the patella (ROCK et al., 2021; GANDOLFI et al., 2018; KARAGIANNIDIS, 2017).
- Semitendinosus (ST): Participant in prone position with the thigh and leg medially rotated. The knee will be flexed to approximately 45°, confirmed with a goniometer. The measurement point will be at 50% of the distance between the ischial tuberosity and the medial epicondyle of the tibia (GANDOLFI et al., 2018; KARAGIANNIDIS, 2017).

Participants will be instructed to keep the leg muscles completely relaxed during the assessment. The evaluated limb will be properly stabilized to minimize any unwanted movement. To ensure consistency between pre- and post-intervention assessments, each measurement site will be marked with a permanent marker and reinforced weekly.

Additionally, to prevent post-exercise edema from affecting the results, images will be acquired 48 to 72 hours before the first session and after the final training session (BRIGATTO et al.,

2022). This timing aligns with previous studies showing that acute increases in MT return to baseline within 48 hours after a training session (OGASAWARA et al., 2012).

Minimal pressure will be applied during measurements to avoid distortion of the underlying tissues. When image quality is deemed satisfactory, three images will be captured per muscle and saved on the device. The average of three different points on each image will later be calculated using ImageJ software (National Institutes of Health).

2.5.4.6 Gait Analysis

The spatiotemporal parameters of gait will be analyzed using the Zeno Walkway (120 Hz), and the data will be processed with ProtoKinetics Movement Analysis Software (PKMAS). The Zeno Walkway is a pressure-sensor mat consisting of 16 levels of sensors distributed over a length of 4.5 meters and a width of 0.6 meters. It comprises two layers: pressure sensors and a protective cover, which will detect and collect pressure data during gait assessments (LYNALL et al., 2017; PROTOKINETICS, 2013). This motion analysis system will allow for the evaluation of various static and dynamic tasks, aiding in the identification of early symptoms of degenerative conditions and diseases.

The Zeno Walkway will be used to evaluate and analyze any condition affecting gait or balance, including neurological conditions (e.g., cerebral palsy, stroke, Parkinson's disease, multiple sclerosis), orthopedic conditions (e.g., injury rehabilitation), geriatric conditions (e.g., fall risk, osteoarthritis, motor deficits, and sensory alterations), as well as the use of prostheses and orthoses (e.g., amputation, prosthetic optimization) and pediatric disorders (e.g., equinus gait, cerebral palsy, and spina bifida) (PADULA et al., 2015).

Spatiotemporal gait parameters will be assessed under two distinct walking conditions: self-selected walking speed (SSWS) and fast walking speed (FWS). Each participant will complete the full course (walking to the end of the walkway and back) five times, and the average of the trials will be used for analysis. An additional 2 meters will be provided before and after the timed portion of the walkway to allow for acceleration and deceleration, ensuring that only steady-state walking velocity is captured for analysis (MACFARLANE; LOONEY, 2008; MIDDLETON et al., 2016).

For the SSWS condition, participants will be instructed to walk at their usual and comfortable pace, as if walking along a sidewalk, at their own rhythm and without rushing (MIDDLETON et al., 2016). For the FWS condition, participants will be instructed to walk as fast as safely possible, as if they were in a hurry to reach a destination, without running (MIDDLETON et al., 2016).

The following gait parameters will be evaluated: step length (the distance between the initial contact point of one foot and the initial contact position of the opposite foot); stride length (the distance from the heel contact of one foot to the subsequent heel contact of the same foot); step width (the perpendicular distance between the line connecting the ipsilateral heel contacts and the contralateral heel contact between these events); step time (the time elapsed for a step, measured from the initial contact of one foot to the next contact of the opposite foot); stride time (the time from the initial contact of one foot to the subsequent contact of the same foot); stance time (the total time during which the foot is in contact with the ground); swing time (the time during which the foot is not in contact with the ground); and, cadence (the number of steps minus one, divided by walking time and converted to steps per minute ($steps \cdot min^{-1}$) (PROTOKINETICS, 2013).

Gait variability, described as the spatial or temporal inconsistency of strides (e.g., time from one foot contact to the next contact of the same foot), will be quantified using the coefficient of variation (CV) (PROTOKINETICS, 2013):

Gait variability (CV) = (Standard Deviation / Mean) x 100%

2.5.5 Access and storage of personal and study data

The data generated from this study will be under the responsibility of the Principal Investigator, Prof. Dr. André Rodacki. All data will be stored in a coded format on his personal computer. The minimum data retention period will be five years. Research assistants may have access to the data. To ensure participant confidentiality, all individuals will be assigned a unique identification code.

2.5.6 Research management

This will be a full-scale interventional study designed to evaluate the effectiveness of a structured training protocol. The management of the study will involve multiple team members

with clearly defined responsibilities to ensure rigorous methodological execution and participant safety. An orthopaedic physician will be responsible for monitoring the safety of participants throughout the intervention period, as well as confirming that all individuals meet the predefined eligibility criteria for inclusion in the training program. The Principal Investigator, Letícia Pophal Cutisque (PhD candidate), will be responsible for participant recruitment and assessments, and direct implementation of the training protocol. The Chief Investigator, Prof. André Rodacki, will supervise all stages of the research process, ensuring adherence to the study design, ethical standards, and data quality. Dr. John Buckley, a senior academic at the School of Engineering, will contribute to the methodological design and will play a central role in the dissemination of findings, assisting in the preparation of scientific manuscripts derived from this research. Letícia Pophal Cutisque is currently a doctoral researcher. Prof. André Rodacki is a permanent faculty member in the Department of Physical Education, with research focused on physical activity and health. Dr. Buckley holds a permanent academic position and brings extensive expertise in biomechanics and applied human movement analysis.

3.0 Dissemination

Although dissemination of findings through high-quality peer-reviewed journals and presentations at international conferences will represent the primary dissemination strategy, results will also be shared with relevant end-users. In addition, study participants will receive summary reports outlining the main findings of the research.

4.0 Project management

The project management and oversight were conducted under the supervision of the Chief Investigator, Prof. André Rodacki. The execution of the experimental procedures was carried out by the Principal Investigator, Prof. Letícia Pophal Cutisque.

5.0 References

AAGAARD, P. et al. Mechanical muscle function, morphology, and fiber type in lifelong trained elderly. Medicine and science in sports and exercise, v. 39, n. 11, p. 1989–96, 2007.

ABE, T. et al. Effects of low-intensity cycle training with restricted leg blood flow on thigh muscle volume and VO2max in young men. Journal of Sports Science and Medicine, v. 9, n.3, p. 452-8, 2010.

ABE, T.; KEARNS, C. F.; SATO, Y. Muscle size and strength are increased following walk training with restricted venous blood flow from the leg muscle, Kaatsu-walk training. Journal of Applied Physiology, v. 100, n. 5, p. 1460–6, 2006.

ANDRADE, S. F. et al. Effects of electrostimulation with blood flow restriction on muscle thickness and strength of the Soleus. Journal of Exercise Physiology Online, v. 19, n. 3, p. 59–70, 2016.

ARHOS, E. K. et al. Quadriceps Strength Symmetry Does Not Modify Gait Mechanics After Anterior Cruciate Ligament Reconstruction, Rehabilitation, and Return-to-Sport Training. The American Journal of Sports Medicine, v. 49, n. 2, p. 417–25, 2021.

ATKINS, S. J. et al. The Presence of Bilateral Imbalance of the Lower Limbs in Elite Youth Soccer Players of Different Ages. Journal of strength and conditioning research, v. 30, n. 4, p. 1007–13, 2016.

BEATO, M. et al. Lower-Limb Muscle Strength, Anterior-Posterior and Inter-Limb Asymmetry in Professional, Elite Academy and Amateur Soccer Players. Journal of Human Kinetics, v. 77, n. 1, p. 135–46, 2021.

BEEKLEY, M. D.; SATO, Y.; ABE, T. KAATSU-walk training increases serum bone-specific alkaline phosphatase in young men. International Journal of KAATSU Training Research, v. 1, n. 2, p. 77–81, 2005.

BENTO, P. C. B. et al. Peak torque and rate of torque development in elderly with and without fall history. Clinical Biomechanics, v. 25, n. 5, p. 450–454, 2010a.

BERG, H. E.; TEDNER, B.; TESCH, P. A. Changes in lower limb muscle cross-sectional area and tissue fluid volume after transition from standing to supine. Acta Physiologica Scandinavica, v. 148, n. 4, p. 379–85, 1993.

BISHOP, C. et al. Interlimb asymmetries: Understanding how to calculate differences from bilateral and unilateral tests. Strength and Conditioning Journal, v. 40, n. 4, p. 1–6,2018.

BISHOP, C.; TURNER, A.; READ, P. Effects of inter-limb asymmetries on physical and sports performance: a systematic review. Journal of Sports Sciences, v. 36, n. 10, p. 1135–44, 2018.

BORG, G. Borg 's Perceived Exertion and Pain Scales. Champaign: Human Kinetics, 1998.

BREIVIK, H. et al. Assessment of pain. British journal of anaesthesia, v. 101, n. 1, p. 17–24, 2008.

BRIGATTO, F. A. et al. High Resistance-Training Volume Enhances Muscle Thickness in Resistance-Trained Men. Journal of strength and conditioning research, v. 36, n. 1, p. 22–30, 2022.

BROWN, H. et al. Factors affecting occlusion pressure and ischemic preconditioning. European journal of sport science, v. 18, n. 3, p. 387–96, 2018.

BROWN, L. E.; WEIR, J. P. ASEP Procedures Recommendation I: Accurate Assessment of Muscular Strength and Power. International Electronic Journal, v. 4, n. 3, p. 1–21, 2001.

CHODZKO-ZAJKO, W. J. et al. Exercise and physical activity for older adults. Medicine and Science in Sports and Exercise, v. 41, n. 7, p. 1510-30, 2009.

CLARKSON, M. J.; CONWAY, L.; WARMINGTON, S. A. Blood flow restriction walking and physical function in older adults: A randomized control trial. Journal of Science and Medicine in Sport, v. 20, n. 12, p. 1041–46, 2017.

COHEN, J. Statistical Power Analysis for the Behavioral Sciences. Second Edition. New York: Academic Press, 1988.

DOTAN, R. et al. Child-adult differences in the kinetics of torque development. Journal of sports sciences, v. 31, n. 9, p. 945–53, 2013.

EKNOYAN, G. Adolphe Quetelet (1796-1874) - The average man and indices of obesity. Nephrology Dialysis Transplantation, v. 23, n. 1, p. 47–51, 2008.

ERELINE, J.; GAPEYEVA, H.; PÄÄSUKE, M. Comparison of twitch contractile properties of plantarflexor muscles in Nordic combined athletes, cross-country skiers, and sedentary men. European Journal of Sport Science, v. 11, n. 1, p. 61–67, 2011.

FALLON, N. E. et al. The impact of cuff width on perceptual responses during and following blood flow restricted walking exercise. Clinical Physiology and Functional Imaging, v. 42, n. 1, p. 29–34, 2022.

FARAS, T. J. et al. The effect of unilateral blood flow restriction on temporal and spatial gait parameters. Heliyon, v. 5, n. 1, 2019.

FATELA, P. et al. Acute effects of exercise under different levels of blood-flow restriction on muscle activation and fatigue. European journal of applied physiology, v. 116, n. 5, p. 985-95, 2016.

GANDOLFI, M. et al. Changes in the sensorimotor system and semitendinosus muscle morphometry after arthroscopic anterior cruciate ligament reconstruction: a prospective cohort study with 1-year follow-up. Knee Surgery, Sports Traumatology, Arthroscopy, v. 26, n. 12, p. 3770–79, 2018.

GILES, L. et al. Quadriceps strengthening with and without blood flow restriction in the treatment of patellofemoral pain: a double-blind randomised trial. British Journal of Sports medicine, v. 51, n. 23, p. 1688–1694, 2017.

GUEDES, D. P.; GUEDES, J. E. R. P. Manual Prático para Avaliação em Educação Física. 1a ed. Barueri - SP: Editora Manole, 2006.

HORTOBÁGYI, T. et al. Old adults perform activities of daily living near their maximal capabilities. Journals of Gerontology - Series A Biological Sciences and Medical Sciences, v. 58, n. 5, p. 453–460, 2003.

HUGHES, L. et al. Blood flow restriction training in clinical musculoskeletal rehabilitation: A systematic review and meta-analysis. British Journal of Sports Medicine, v. 51, n. 13, p. 1003-11, 2017.

KARAGIANNIDIS, E. Semitendinosus muscle architecture during maximum isometric contractions in individuals with anterior cruciate ligament reconstruction and controls. Muscle, Ligaments and Tendons Journal, v. 7, n. 1, p. 147, 2017.

KARGARAN, A. et al. Effects of dual-task training with blood flow restriction on cognitive functions, muscle quality, and circulatory biomarkers in elderly women. Physiology & Behavior, v. 239, p. 113500, 2021.

KILGAS, M. A. et al. Exercise with Blood Flow Restriction to Improve Quadriceps Function Long After ACL Reconstruction. International Journal of Sports Medicine, v. 40, n. 10, p. 650–656, 2019.

KYRITSIS, P. et al. Likelihood of ACL graft rupture: not meeting six clinical discharge criteria before return to sport is associated with a four times greater risk of rupture. British Journal of Sports Medicine, v. 50, n. 15, p. 946–951, 2016.

LAURENTINO, G. et al. Effects of strength training and vascular occlusion. International Journal of Sports Medicine, v. 29, n. 8, p. 664–667, 2008.

LIXANDRÃO, M. E. et al. Effects of exercise intensity and occlusion pressure after 12 weeks of resistance training with blood-flow restriction. European journal of applied physiology, v. 115, n. 12, p. 2471–2480, 2015.

LIXANDRÃO, M. E. et al. Magnitude of Muscle Strength and Mass Adaptations Between High-Load Resistance Training Versus Low-Load Resistance Training Associated with Blood-Flow Restriction: A Systematic Review and Meta-Analysis. Sports Medicine, v. 48, n. 2, p. 361–378, 2018.

LOENNEKE, J. P. et al. Potential safety issues with blood flow restriction training. Scandinavian Journal of Medicine and Science in Sports, v. 21, n. 4, p. 510-8, 2011.

LYNALL, R. C. et al. Reliability and validity of the protokinetics movement analysis software in measuring center of pressure during walking. Gait & Posture, v. 52, p. 308–311, 2017.

MACFARLANE, P. A; LOONEY, M. A. Walkway Length Determination for Steady State Walking in Young and Older Adults. Research Quarterly for Exercise and Sport, v. 79, n. 2, p. 261–267, 2008.

MATSUDO, S. et al. Questionário Internacional de Atividade Física (IPAQ): Estudo de Validade e Reprodutibilidade no Brasil. Revista Brasileira de Atividade Física e Saúde, v. 6, n. 2, 2001.

MIDDLETON, A. et al. Self-selected and maximal walking speeds provide greater insight into fall status than walking speed reserve among community-dwelling older adults. American Journal of Physical Medicine and Rehabilitation, v. 95, n. 7, p. 475–482, 2016.

MOK, E. et al. Negative effects of blood flow restriction on perceptual responses to walking in healthy young adults: A pilot study. Heliyon, v. 6, n. 8, 2020.

OGASAWARA, R. et al. Time course for arm and chest muscle thickness changes following bench press training. Interventional Medicine & Applied Science, v. 4, n. 4, p. 217, 2012.

OZAKI, H. et al. Increases in Thigh Muscle Volume and Strength by Walk Training with Leg Blood Flow Reduction in Older Participants. The Journals of Gerontology Series A: Biological Sciences and Medical Sciences, v. 66A, n. 3, p. 257–263, 2011.

OZAKI, H. et al. Effects of 10 weeks walk training with leg blood flow reduction on carotid arterial compliance and muscle size in the elderly adults. Angiology, v. 62, n. 1, p. 81–86, 2011.

OZEMEK, C.; LAVIE, C. J.; ROGNMO, O. Global physical activity levels - Need for intervention. Progress in Cardiovascular Diseases, v. 62, n. 2, p. 102–107, 2019.

PADULA, W. V. et al. Risk of fall (RoF) intervention by affecting visual egocenter through gait analysis and yoked prisms. NeuroRehabilitation, v. 37, n. 2, p. 305–314, 2015.

PARKINSON, A. O. et al. The Calculation, Thresholds and Reporting of Inter-Limb Strength Asymmetry: A Systematic Review. Journal of Sports Science and Medicine, p. 594–617, 2021.

PAWLOWSKI, B. et al. Human body symmetry and immune efficacy in healthy adults. American journal of physical anthropology, v. 167, n. 2, p. 207–216, 2018.

PRATT, M. et al. Attacking the pandemic of physical inactivity: what is holding us back? British journal of sports medicine, v. 54, n. 13, p. 760–762, 2020.

PROTOKINETICS. ProtoKinetics Movement Analysis Software: Measurements and Definitions. Havertown, 2013.

ROCK, K. et al. Assessing the Reliability of Handheld Dynamometry and Ultrasonography to Measure Quadriceps Strength and Muscle Thickness in Children, Adolescents, and Young Adults. Physical & occupational therapy in pediatrics, v. 41, n. 5, p. 540–554, 2021.

ROHMAN, E.; STEUBS, J. T.; TOMPKINS, M. Changes in involved and uninvolved limb function during rehabilitation after anterior cruciate ligament reconstruction: implications for Limb Symmetry Index measures. The American journal of sports medicine, v. 43, n. 6, p. 1391–1398, 2015.

SARABON, N. et al. Factors influencing bilateral deficit and inter-limb asymmetry of maximal and explosive strength: motor task, outcome measure and muscle group. European Journal of Applied Physiology, v. 120, n. 7, p. 1681–1688, 2020.

SCHULZ, K. F.; ALTMAN, D. G.; MOHER, D. CONSORT 2010 Statement: Updated guidelines for reporting parallel group randomised trials. BMJ (Online), v. 340, n. 7748, p. 698–702, 2010.

SHARMA, V. et al. Characterization of acute ischemia-related physiological responses associated with remote ischemic preconditioning: a randomized controlled, crossover human study. Physiological reports, v. 2, n. 11, 2014.

SKIBA, G. H.; ANDRADE, S. F.; RODACKI, A. F. Effects of functional electro-stimulation combined with blood flow restriction in affected muscles by spinal cord injury. Neurological sciences: official journal of the Italian Neurological Society and of the Italian Society of Clinical Neurophysiology, v. 43, n. 1, p. 603–613, 2022.

SMAJLA, D.; ŽITNIK, J.; ŠARABON, N. Investigation of inter-limb symmetry in knee extensors using different strength outcome measures. Diagnostics, v. 11, n. 10, 2021.

THOMAS, J. R.; NELSON, J. K.; SILVERMAN, S. J. Métodos de pesquisa em atividade física. 6a ed. Artmed, 2012.

TICINESI, A. et al. Assessing sarcopenia with vastus lateralis muscle ultrasound: an operative protocol. Aging clinical and experimental research, v. 30, n. 12, p. 1437–1443, 2018.

VECHIN, F. C. et al. Comparisons between low-intensity resistance training with blood flow restriction and high-intensity resistance training on quadriceps muscle mass and strength in elderly. Journal of strength and conditioning research, v. 29, n. 4, p. 1071–76, 2015.

WORLD HEALTH ORGANIZATION. Infection prevention and control during health care when COVID-19 is suspected. Interim Guid , p. 1–5, 2020.

XU, J.; HUG, F.; FU, S N.. Stiffness of individual quadriceps muscle assessed using ultrasound shear wave elastography during passive stretching. Journal of Sport and Health Science, v. 7, n. 2, p. 245–249, 2018.
